# An acentrosomal aster with atypical microtubule polarity recruits cytokinesis signals to its center in *Xenopus* egg extracts

**DOI:** 10.1242/jcs.263766

**Published:** 2025-09-26

**Authors:** Ling Jin, Muchen Liu, Xianrui Cheng

**Affiliations:** Section of Molecular and Computational Biology, Department of Biological Sciences, University of Southern California, Los Angeles, CA 90089, USA

**Keywords:** Self-organization, Microtubules, *Xenopus* egg extracts, Motors, Cytokinesis, Aster

## Abstract

*Xenopus* egg extracts can self-organize into cell-like compartments without the classic microtubule organizer centrosome. Compartment formation requires microtubules, but the organization of microtubules throughout the process remains unclear. Here, we show that the earliest organized microtubule structures to emerge during cell-like compartment formation are centrosome-independent asters. In contrast to the microtubule orientation of a centrosome-nucleated aster, most microtubules in the centrosome-independent aster point their plus ends toward the center. Formation of these asters requires the microtubule motor MKLP2 (also known as KIF20A) and Aurora kinase B activity. The aster center accumulates microtubule plus end-binding protein EB1–GFP and the plus end-tracking motor kinesin-1–GFP, and also recruits cytokinesis-related proteins GFP–MKLP1 (KIF23), active RhoA, and F-actin. Together, our findings identify an early microtubule structure in cell-like compartment self-organization and link it to the cytokinesis pathway.

## Teaser

**Summary:** We report a centrosome-independent, self-organizing microtubule aster that exhibits mostly inverted microtubule polarity and recruits cytokinesis signals to its center.

## INTRODUCTION

The cytoplasm is organized into highly ordered spatial patterns that are important for cellular functions. Reconstitution of these structures *in vitro* has proven to be a productive way to understand how the organized patterns work in cells (Dogterom and Surrey, 2013). A reconstitution model system that recapitulates the composition, physical properties and biological functions of the cytoplasm is the cell-free *Xenopus laevis* (African clawed frog) egg extract (Chang and Ferrell, 2013, 2018; Field and Mitchison, 2018; Field et al., 2017; Mitchison and Field, 2021; Murray, 1991; Newmeyer et al., 1994; Nguyen et al., 2014; Valentine et al., 2005). An egg extract is a pool of undiluted cytoplasmic components, including proteins, nucleic acids, metabolites and organelles, that can self-organize into spatial patterns seen in living cells. Extracts can be biochemically manipulated and imaged live with fluorescence microscopy, making them an excellent experimental model for dissecting the mechanisms of cytoplasmic organization (Cheng and Ferrell, 2019, 2021; Deming and Kornbluth, 2006; Field et al., 2017; Murray, 1991). Many essential subcellular organizations have been reconstituted using egg extracts, such as the mitotic spindle (Heald et al., 1996; Sawin and Mitchison, 1991), the cytokinesis machinery (Landino et al., 2021; Nguyen et al., 2014) and microtubule asters (Félix et al., 1994; Field and Mitchison, 2018). More recently, it has been discovered that larger-scale cell-like compartments capable of many rounds of mitoses can self-assemble in *Xenopus* egg extracts (Cheng and Ferrell, 2019). These cell-like compartments are similar to the cytoplasmic partitions observed before cytokinetic cleavage of early *Xenopus* embryos (Field et al., 2015; Nguyen et al., 2014; Wühr et al., 2010), but how they self-assemble and their relationship with cytokinesis have yet to be explored.

Cell-like compartment formation depends on microtubules and ATP but not F-actin (Cheng and Ferrell, 2019). Compartments form during the interphase of the cell cycle in plain egg extracts. Plain egg extracts do not contain added *Xenopus* sperm nuclei and their accompanying centrosomes. As a result, components that otherwise localize to the nucleus are present in the cytoplasm in these extracts. Microtubules in extracts are initially depolymerized due to the low temperatures during preparation (0–4°C), and extract components are uniformly distributed in space (Breton and Brown, 1998; Cheng and Ferrell, 2019; Deming and Kornbluth, 2006; Li and Moore, 2020; Murray, 1991; Tilney and Porter, 1967). Self-assembly is initiated when the extracts are incubated at room temperature (∼22°C), which enables microtubule repolymerization. The initial spatial homogeneity is disrupted by the emergence of circular microtubule-depleted zones at random locations, each with a bright microtubule focus at the center. Subsequently, these microtubule-depleted zones expand, merge and morph into the borders that delineate the microtubule-enriched cell-like compartments. The microtubule foci persist during the pattern formation process and remain localized inside the border zones (Cheng and Ferrell, 2019).

Focused microtubule structures and microtubule-depleted zones have been previously observed in egg extracts in other contexts. One type of focused microtubule structure is the center of a microtubule aster. In interphase and mitotic *Xenopus* egg extracts, microtubule asters can be nucleated by centrosomes (Félix et al., 1994; Stearns and Kirschner, 1994). In mitotic extracts, asters can be organized by the microtubule motor dynein after microtubules form in the presence of the microtubule-stabilizing drug Taxol or the guanosine triphosphatase (GTPase) Ran (Ran-GTP) (Ohba et al., 1999; Verde et al., 1991; Wilde and Zheng, 1999). These classic asters share the same microtubule polarity where minus ends point toward the aster center and plus ends point outward. Another type of focused microtubule structure is the antiparallel microtubule bundle in the division plane during cytokinesis. When the expanding edges of two microtubule asters meet, astral microtubules interdigitate and form antiparallel plus end bundles that recruit cytokinesis signal proteins Aurora kinase B, MKLP1 (also known as KIF23) and MKLP2 (also known as KIF20A) (Nguyen et al., 2014). The bundles reduce microtubule density in their vicinity in an Aurora kinase B-dependent manner, giving rise to a straight microtubule-depleted border zone midway between the aster centers (Nguyen et al., 2014). These bundles recruit F-actin, an essential component of the cytokinetic contractile ring that brings about the cleavage furrow. The bundles also recruit active RhoA (RhoA-GTP), a small GTPase that promotes contractile ring formation (Wagner and Glotzer, 2016), to the plasma membrane adjacent to the bundle (Nguyen et al., 2014). Microtubule-depleted zones have also been observed in extracts (Field et al., 2019; Nguyen et al., 2014). Beads coated with Aurora kinase B generate circular microtubule-depleted zones around them (Field et al., 2019), similar to the circular zones observed around the microtubule foci (Cheng and Ferrell, 2019). Despite the apparent similarities to these previously observed microtubule focal structures, the identity of the microtubule foci that emerge in centrosome-free extracts during cell-like compartment formation remains to be determined.

Here, we combine time-lapse live imaging and biochemical methods to characterize the microtubule foci observed in cell-like compartment self-assembly and explore their connection to cytokinesis.

## RESULTS

### Centrosome-independent microtubule asters emerge during the early stage of pattern formation in *Xenopus* egg extracts

**Fig. 1.**
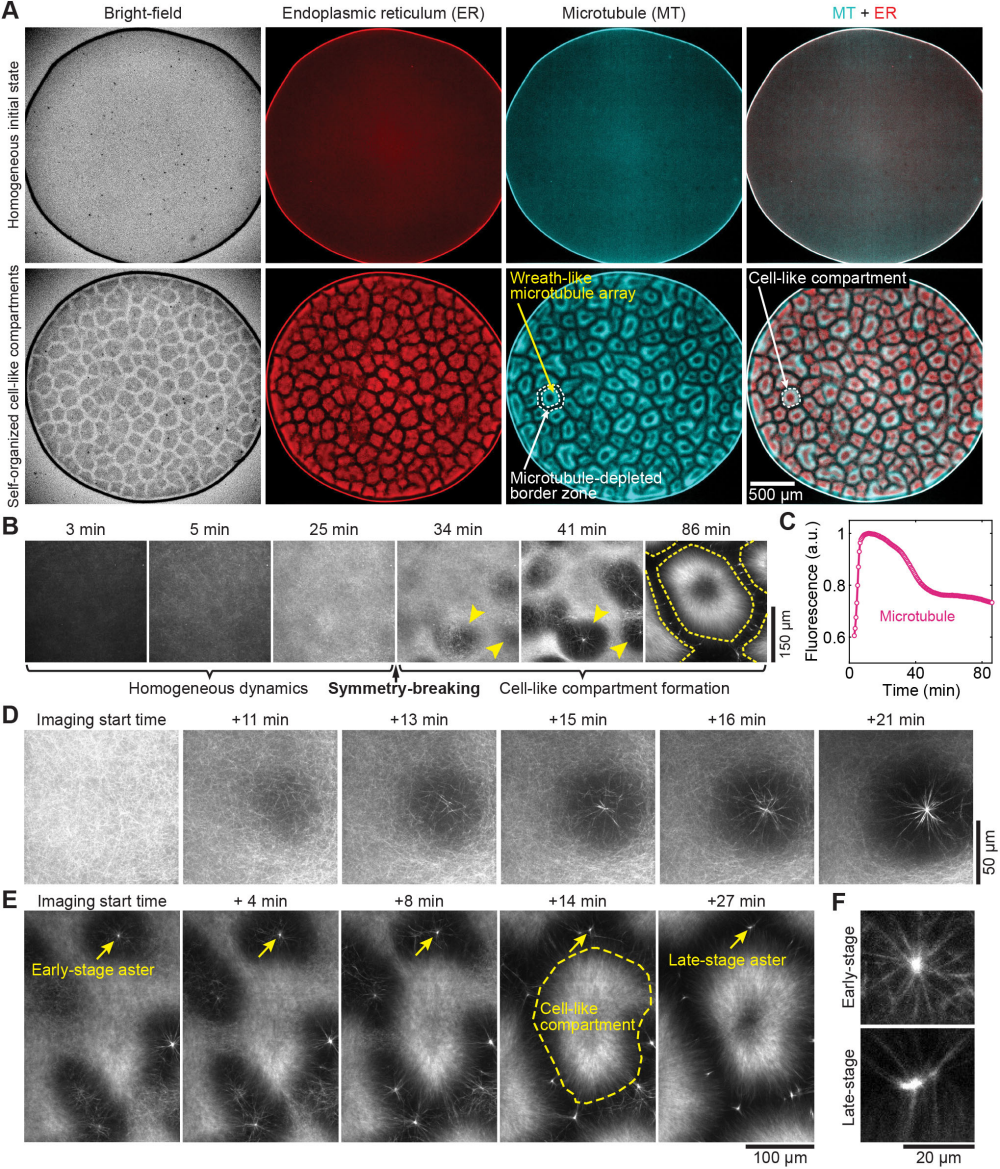
**Centrosome-independent microtubule asters participate in symmetry breaking during pattern formation in *Xenopus* egg extracts.** (A) Self-organization of cell-like compartments in *Xenopus* egg extracts. The round region that occupies most of the field of view is the extract droplet (see Fig. S1A for imaging setup schematics). Bright-field and epifluorescence images were taken at an early time point when the extracts appeared homogeneous (top row) and at a later time point (∼60 min later) when cell-like compartments had formed (bottom row). In the microtubule column, the yellow arrow indicates the wreath-like microtubule array formed within a cell-like compartment. The area between the two white dashed lines just outside the wreath-like microtubule array is a microtubule-depleted zone that defines the borders between compartments. In the MT+ER column, the white dashed line indicates the boundary of a cell-like compartment. Number of independent experiments, *n*>100. (B) Confocal time-lapse montage of microtubule dynamics during self-organization. Imaging started at ∼3 min after the extracts were moved from an ice-water bath to a room temperature environment. The yellow arrowheads at 34 min indicate exemplary microtubule-depleted regions. Yellow arrowheads at 41 min indicate the microtubule-depleted regions in which microtubule foci had emerged. Yellow outlines at 86 min indicate the boundaries of cell-like compartments. Each image is a maximum-intensity projection of 17 confocal planes spanning 32 µm of depth. (C) Quantification of microtubule (visualized by SiR-tubulin) fluorescence intensity for the full time-lapse image series from the experiment shown in B. Each point on the plot indicates the total fluorescence intensity of all 17 confocal planes at the corresponding time point. Fluorescence intensity is measured in arbitrary units (a.u.). (D) Time-lapse montage of microtubule dynamics showing the emergence of a self-organized centrosome-independent aster. Each image is a maximum-intensity projection of three confocal planes spanning 4 µm of depth. The first image on the left was taken at an arbitrary time point when the microtubules were spatially homogenously distributed in the extract. Subsequent images were taken at time increments from the first time point indicated by the text labels (+11 min, +13 min, and so on). (E) Time-lapse montage of microtubule dynamics, showing the asters throughout the cell-like compartment formation process. Each image is a maximum-intensity projection of 12 confocal planes spanning 22 µm of depth. The first image on the left was taken at an arbitrary time point when asters had just formed. Subsequent images were taken at time increments from the first time point indicated by the text label above (+4 min, +8 min, and so on). The yellow arrows track the same aster through time. The yellow dashed line marks a cell-like compartment. (F) Confocal images of early-stage and late-stage asters, showing different morphological features in astral microtubule organization. The top panel is a magnified view of the early-stage aster indicated by the yellow arrow in the first image panel (imaging start time) in E, and the bottom panel is the late-stage aster indicated by the yellow arrow in the last image panel (+27 min) in E. Data presented in B–F are representative of 38 samples across nine independent experiments.

We first repeated the cell-like compartment formation experiment to make sure we could reproduce the phenomenon described previously (Cheng and Ferrell, 2019). We prepared interphase extracts and imaged them with time-lapse microscopy (Cheng and Ferrell, 2019; Cheng and Ferrell, 2021) (Fig. 1A). Note that our extracts allowed F-actin formation as we did not supplement them with actin polymerization inhibitor cytochalasin B. SiR-tubulin, a probe that fluoresces only when bound to polymerized tubulin (microtubules), was added to the extracts to visualize microtubules (Cheng and Ferrell, 2019; Lukinavičius et al., 2014). To track the dynamics of a representative membrane organelle, ER-Tracker, a probe for visualizing the endoplasmic reticulum (ER), was also added. We confirmed that, starting from a spatially homogeneous state without added centrosomes or chromatin, cell-like compartments self-assembled at room temperature (Fig. 1A; Movie 1). The cell-like compartments were separated by microtubule-depleted border zones (Fig. 1A). Each compartment contained a wreath-like microtubule array and an ER-filled body, consistent with previous observations (Cheng and Ferrell, 2019). Only extracts that successfully assembled cell-like compartments were included in our analysis (the number of successful independent experiments was more than 100; the rate of successful assembly was ∼90% in our hands).

To determine how microtubules self-organized into compartments, we imaged extracts shortly after they were subject to room temperature incubation. Using confocal microscopy, we found that microtubules were initially mostly depolymerized, as indicated by low SiR-tubulin fluorescence at the beginning (Fig. 1B, 3 min). Interestingly, the formation of new microtubules occurred uniformly in space and did not break symmetry (Fig. 1B, 3 min to 25 min; Movie 2). Quantification of SiR-tubulin fluorescence showed that the total amount of microtubules kept increasing during this time (Fig. 1C; observations consistent across 14 independent experiments). These observations were corroborated by epifluorescence microscopy data, where microtubules throughout the entire depths of the extract samples were imaged and quantified (Fig. S1B,C; Movie 3; observations consistent across four independent experiments). To determine whether microtubule polymerization sites are also evenly distributed in space, we imaged the growing plus ends of microtubules using confocal microscopy. To visualize actively growing plus ends, we added purified End-binding protein 1 fused with a green fluorescent protein (EB1–GFP) to the extract. EB1–GFP is a recombinant protein that binds specifically to the growing plus ends of microtubules and is commonly used to track them (Bieling et al., 2008; Dixit et al., 2009; Mimori-Kiyosue et al., 2000). Consistent with our earlier observations, the sites of growing plus ends increased uniformly in space during the initial period of self-organization (Fig. S1D; consistent across six independent experiments). As the microtubules self-organized into compartments, EB1–GFP also became spatially patterned and mostly appeared inside the compartments (Fig. S1E; Movie 4; observed in seven independent experiments). These observations suggest that the one-pot self-organization reaction begins with a period of spatially uniform microtubule polymerization, and that these early reactions do not add apparent asymmetric spatial organization. Symmetry breaking in microtubule organization began with the emergence of microtubule-depleted zones (Fig. 1B, 34 min; Movie 2). As these circular zones enlarged, bright microtubule foci became visible at their centers (Fig. 1B, 41 min; Movie 2). These expanding zones eventually met one another and morphed into the borders between cell-like compartments (Fig. 1B, 86 min; Movie 2). Imaging at higher spatiotemporal resolution revealed that as the microtubule-depleted zones emerged, some microtubules persisted inside the zone and rapidly organized into an aster (Fig. 1D). The foci at the center of the zones were the centers of these asters. The asters persisted as the zones transitioned into the borders between cell-like compartments (Fig. 1E; Fig. S2A). The astral microtubules appeared more radially symmetric in early-stage asters than late-stage asters (Fig. 1F; Fig. S2B,C). These observations were consistent across 38 samples from nine independent experiments. Therefore, microtubules self-organize into asters and break the initial spatial symmetry in extracts during cell-like compartment formation. In animal cells, microtubule asters are typically formed by centrosomes (Schwarz, 2021; Urbani and Stearns, 1999). However, *Xenopus* egg extracts do not contain centrosomes (Wiese et al., 2010), and the asters observed here apparently formed without them. We therefore refer to these asters as noncanonical asters in the present study.

When egg extracts are imaged in a sealed chamber (Fig. S1A), oxygen will be depleted by mitochondria even though ATP can still be produced by anaerobic metabolism (Field et al., 2017; Niethammer et al., 2008). To determine whether noncanonical asters and cell-like compartments form when extracts have oxygen supply, we provided extracts with fresh air by exposing the extract droplet to the atmosphere through two small openings in the imaging spacer. Both the noncanonical asters and cell-like compartments formed in extracts in the presence of oxygen supply (Fig. S2D; Movie 5; observed in three independent experiments). We also note that the plain extracts do not contain nuclei, so factors normally localized to the nucleus are present in the cytoplasm and can affect noncanonical aster formation. To determine whether the noncanonical asters and cell-like compartments form when nuclei are present, we added a small amount of demembranated *Xenopus* sperm nuclei to the plain extracts. Both the noncanonical asters and cell-like compartments formed in nuclei-free regions in these extracts despite sharing the cytoplasm with nuclei (Fig. S2E; Movie 6; observed in eight independent experiments).

### Microtubule orientation in the noncanonical aster differs from that in a centrosome aster

**Fig. 2.**
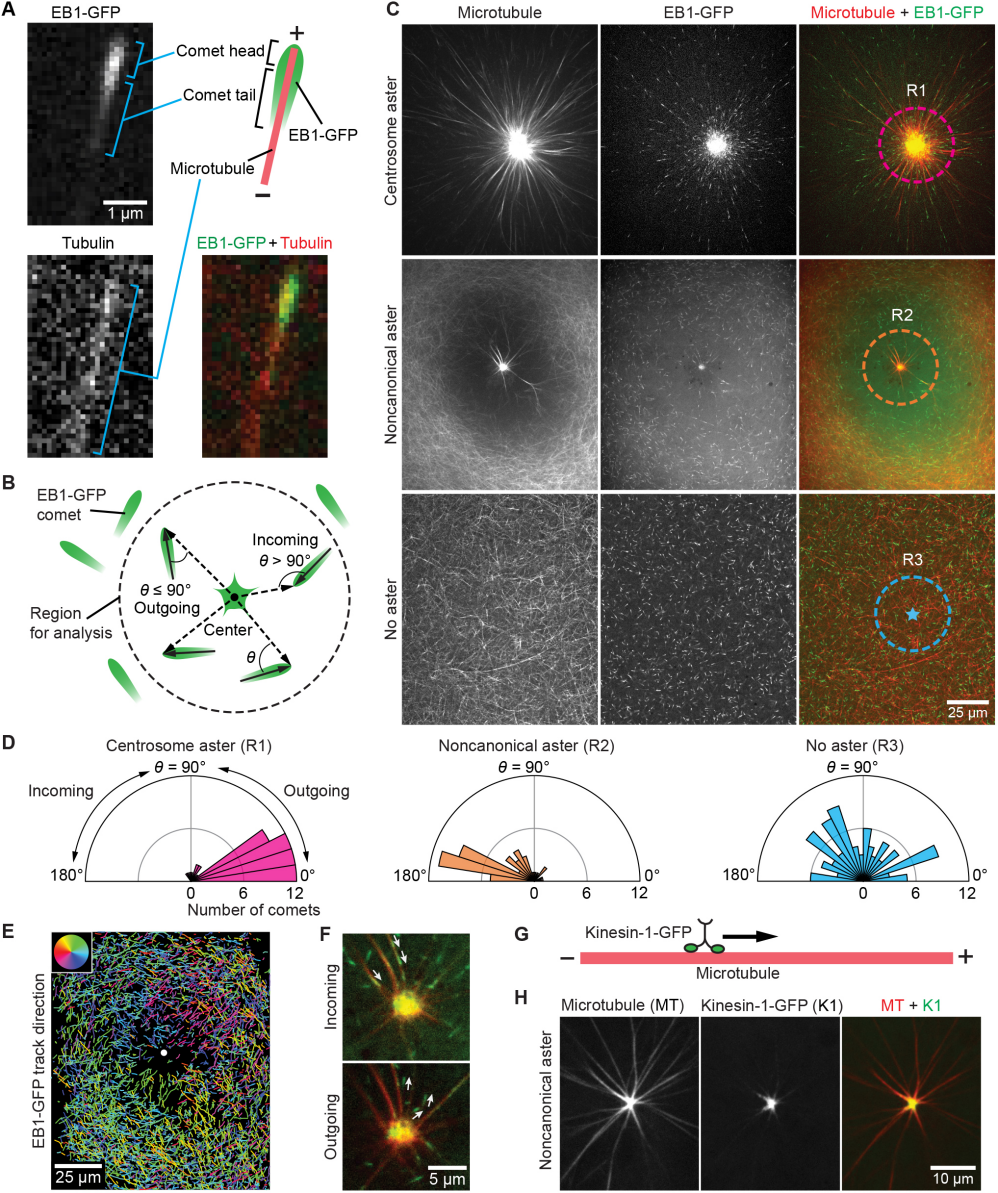
**Microtubule orientation in the noncanonical aster differs from that in a centrosome aster.** (A) Confocal images and schematic representation of the microtubule plus end architecture. (B) Schematic diagram describing the method for determining microtubule orientation relative to a center using EB1–GFP image data. The solid arrows represent comet vectors, and the dashed arrows represent radial vectors. The dashed circle marks the region of analysis. Only the EB1–GFP comets whose heads fall into this region are analyzed to determine the orientation of their associated microtubules. (C) Single-plane confocal (optical section thickness ∼1.3 µm) images of microtubule and EB1–GFP spatial patterns in a centrosome aster, a noncanonical aster and a region with no aster. The EB1–GFP images are used to determine the orientation of growing microtubules using the method described in B, in the main text and in Fig. S3. The dashed circles indicate the regions of analysis as defined in B, for regions where there is a centrosome aster (R1), a noncanonical aster (R2) and for a region with no asters (R3). For R1 and R2, the geometric center of the EB1–GFP enriched region at the aster center is used as the circle center. The circle radius for both is 22 µm. For R3, an arbitrary point in the middle of the EB1–GFP image is chosen as the circle center, and the circle radius is also 22 µm. (D) Polar histograms showing the distributions of the angle metric *θ* for the EB1–GFP comets in R1, R2 and R3 shown in C. *θ*>90° means that the microtubules the comets are associated with point their plus ends toward the center, and *θ*<90° means the plus ends point away from the center. In the histogram for the centrosome aster (R1), counts beyond 12 are not displayed to keep the axis limits the same as R2 and R3, without affecting the conclusions. Number of independent experiments, *n*=2 for the centrosome aster (R1), *n*=4 for the noncanonical aster (R2) and *n*=3 for the no aster case (R3). (E) Trajectories of EB1–GFP comet movement over 150 s from the experiment shown in C. The color of a track indicates its average direction (see Materials and Methods). The color for each direction is given by the color disk at the top-left corner. For example, if a track on average goes perpendicularly toward the left edge of the image, its color will be red. The center of the noncanonical aster is indicated by a white dot. (F) Single-plane confocal images showing EB1–GFP orientation near the center of the noncanonical aster. The white arrows indicate the direction the EB1–GFP comet head is pointing to. The validity of the direction is verified by time-lapse imaging data for each arrow-marked comet. (G) Schematic diagram of a microtubule plus end-directed motor kinesin-1–GFP walking on a microtubule. (H) Single-plane confocal images showing steady state localization of human kinesin-1–GFP in egg extracts. Kinesin-1–GFP accumulates at the center of the noncanonical aster. Images shown are representative of 39 noncanonical asters from ten independent experiments.

To better understand the spatial organization of the noncanonical aster, we examined the microtubule orientation relative to the aster center using EB1–GFP. EB1 protein (also known as MAPRE1) coats a segment of the microtubule and appears as a moving comet (Akhmanova and Steinmetz, 2008; Bieling et al., 2008; Mimori-Kiyosue et al., 2000). The head of the comet marks the tip of the plus end, and the tail extends toward the direction of the minus end (Tamura and Draviam, 2012), as shown in Fig. 2A. Therefore, microtubule orientation relative to the aster center can be quantified using an angle metric *θ* calculated from EB1–GFP comet images (Fig. 2B; Fig. S3A–C; see details in Materials and Methods).

To test this approach, we quantified microtubule orientation in centrosome asters, where microtubules are known to point plus ends away from the aster center (Alberts et al., 2022; Ishihara et al., 2014; Lüders and Stearns, 2007). EB1–GFP comets were observed throughout the aster and were enriched at the aster center (Fig. 2C, centrosome aster panels; Movie 7). Most of the growing microtubules (86/89, 97%) near the aster center pointed plus ends away by our *θ* metric (Fig. 2D, centrosome aster plot; Fig. S3D, centrosome aster panels; observed in two independent experiments), thus validating our approach.

We then determined the orientation of growing microtubules in the noncanonical aster. We found that both EB1–GFP and microtubules were enriched at the center of the aster (Fig. 2C, noncanonical aster panels; Movie 8; observed for 47 out of 57 asters from seven independent experiments). Notably, the enrichment of EB1–GFP at the center occurred after that of microtubules (Fig. S4A; observed for 41 asters in two independent experiments). Most of the growing microtubules (43/49, 88%) near the center pointed plus ends toward the center (Fig. 2D, noncanonical aster plot; Fig. S3D, noncanonical aster panels; observed for 13 asters in four independent experiments; the average percentage of centripetal microtubules from these experiments was 79%, with s.e.m. of 2%), in contrast to their centrifugal orientation in the centrosome aster. A map of EB1–GFP comet movement trajectories around the noncanonical aster is presented in Fig. 2E. Many of the centripetal EB1–GFP comets moved along the thick astral microtubule bundles that connected to the EB1–GFP-enriched aster center (Fig. 2F; Fig. S4B,C; Movie 9; observed in 13 out of 15 asters from four independent experiments), with some of them appearing to merge into the center (Movie 9). A small number of comets emanated from the aster center (Fig. 2F; Fig. S4B; Movie 9; observed in 13 out of 15 noncanonical asters from four independent experiments). These dynamics were also observed in noncanonical asters after cell-like compartments had formed (Fig. S4C). Note that we could not determine whether the EB1–GFP comets in the noncanonical aster region belonged to the microtubules of the noncanonical aster.

When no asters were present, no EB1–GFP-enriched focal regions were observed and all EB1–GFP appeared as comets (Fig. 2C, no aster panels). Microtubule orientation appeared random (Fig. 2D, no aster plot; three independent experiments). The average percentage of centripetal EB1–GFP comets across all experiments was 51% (s.e.m. 2%), as expected from random comet orientations. We found that ∼86% (s.e.m. 4%) of the EB1–GFP comets terminated at or near the center, and 14% (s.e.m. 4%) emanated from the center (calculated from 11 asters across four biological repeats; see Materials and Methods for details). We conclude that in the noncanonical aster, most growing microtubules orient their plus ends toward the aster center.

The centripetal orientation of growing microtubules and the enrichment of EB1–GFP at the aster center raise the possibility that the noncanonical aster focuses microtubule plus ends at its center. To test this possibility, a recombinant human kinesin-1 protein fused with GFP (kinesin-1–GFP; Woehlke et al., 1997) was used to indicate the polarity of microtubules. Kinesin-1 is a classic microtubule motor that walks towards the plus ends of microtubules, and its recombinant GFP fusion proteins have been widely used to indicate microtubule polarity (Niedzialkowska et al., 2024; Tas et al., 2017) (Fig. 2G). Purified human kinesin-1–GFP and SiR-tubulin were added to extracts and imaged with confocal microscopy. Kinesin-1–GFP moved along microtubules, indicating that the human kinesin-1–GFP protein was functional in *Xenopus* egg extracts (Movie 10). Kinesin-1–GFP was enriched at the center of the noncanonical aster, suggesting that the center predominantly focuses the plus ends of astral microtubules (Fig. 2H; Fig. S5A,B; Movie 11). The localization pattern persisted even after the cell-like compartment assembly was completed (Fig. S5C,D). This kinesin-1–GFP localization pattern was consistent across 39 noncanonical asters from ten independent experiments. We did not detect appreciable kinesin-1–GFP accumulation at the center of centrosome asters, whose microtubule plus ends point outward from the center (Fig. S5E; observed in two independent experiments). Thus, the noncanonical aster center is a microtubule plus end hub, in contrast to the centrosome aster center which is a minus end hub.

To estimate the tubulin populations that might have contributed to noncanonical asters and cell-like compartments, we added fluorescently labeled tubulin protein to egg extracts and quantified tubulin fluorescence inside and outside the compartments (see Fig. S5F for an example of how compartments were identified from images). We found that 82% of the total tubulin fluorescence was inside compartments and 18% outside (s.e.m. 2%; calculated from four independent experiments).

### Formation of the noncanonical aster requires the microtubule motor MKLP2 and Aurora kinase B activity

In the absence of centrosomes, polarity-inverted microtubule asters can be organized by plus end-directed microtubule motors *in vitro* (Nédélec et al., 2001; Nitta et al., 2021; Surrey et al., 2001; Urrutia et al., 1991). To understand how the noncanonical aster formed in egg extracts, we assessed the roles of two candidate plus end-directed motors: the kinesin-6 family member MKLP2 and the kinesin-5 family member Eg5 (also known as KIF11).

**Fig. 3.**
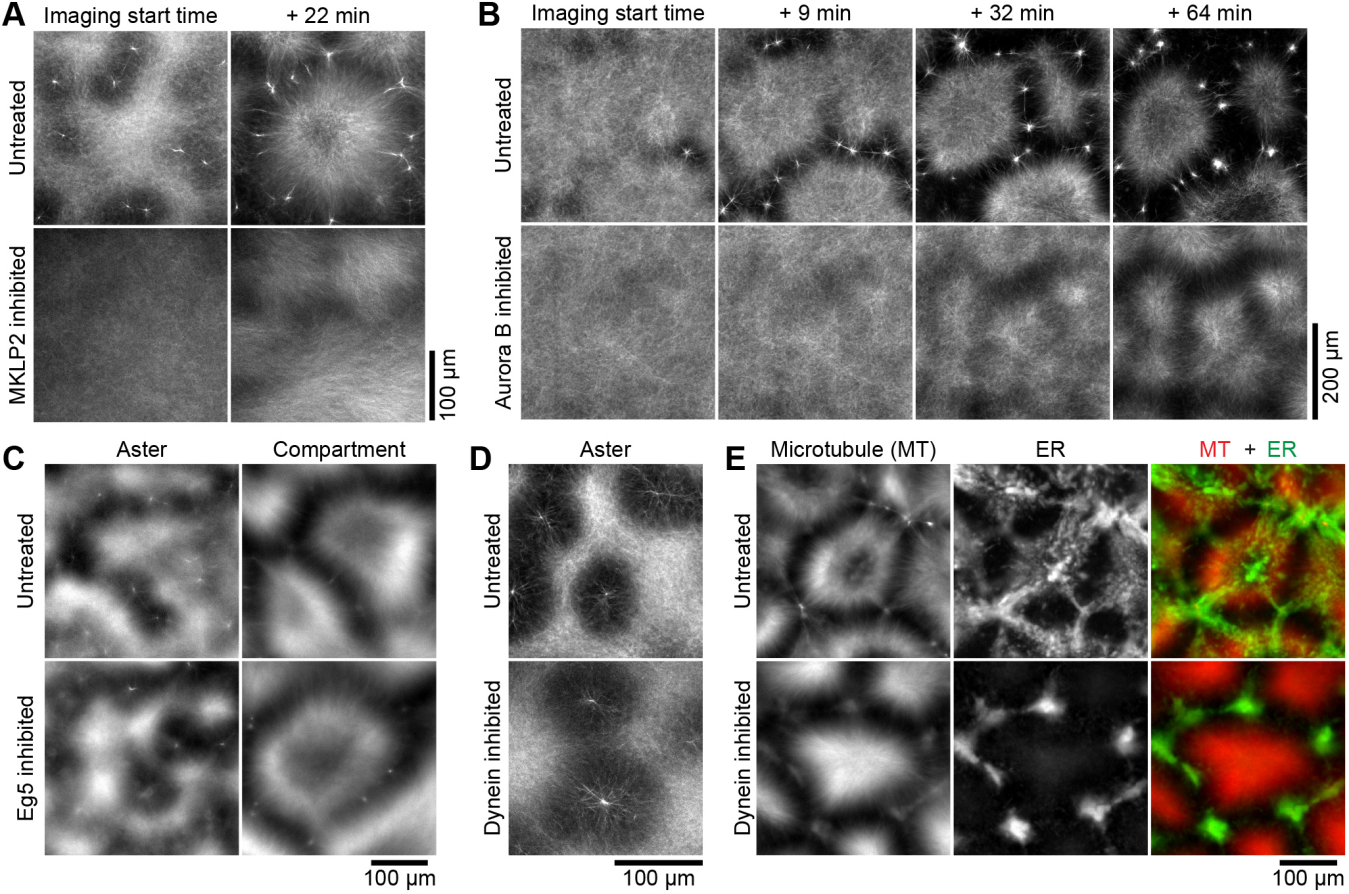
**Formation of the noncanonical aster requires the microtubule motor MKLP2 and Aurora kinase B activity.** (A) Confocal images of microtubule organization in control extracts and extracts with 100 µM MKLP2 inhibitor paprotrain. *n*=4. Each image is a maximum-intensity projection of nine confocal planes spanning 24 μm of depth. (B) Confocal images of microtubule dynamics in control extracts (top row) and extracts with 40 µM Aurora kinase B inhibitor barasertib (bottom row). *n*=6. Each image is a maximum-intensity projection of nine confocal planes spanning 16 µm of depth. For both A and B, imaging started at an arbitrary time point when asters had just begun to form in the untreated extracts. (C) Widefield epifluorescence images of microtubule organization in control extracts and extracts with 100 µM kinesin Eg5 inhibitor STLC. *n*=10. (D) Confocal images of microtubule organization in control extracts and extracts with 2 µM dynein inhibitor GST–p150-CC1. *n*=6. (E) Widefield epifluorescence images of microtubule and ER organization in control extracts and extracts with 0.68 µM GST–p150-CC1. *n*=2.

Inhibition of MKLP2 using the selective inhibitor paprotrain (Tcherniuk et al., 2010) abolished noncanonical asters and cell-like compartments (Fig. 3A; observed in four independent experiments), suggesting that MKLP2 is required for noncanonical aster and cell-like compartment formation. At anaphase onset, MKLP2 transports the chromosomal passenger complex (CPC) from the centromeres to the central spindle (Adriaans et al., 2020; Carmena et al., 2012; Gruneberg et al., 2004). Aurora kinase B is a core component of the CPC and a cargo of MKLP2 (Gruneberg et al., 2004). We thus wondered whether Aurora kinase B is required for noncanonical aster formation. Inhibition of Aurora kinase B with the selective inhibitor barasertib (Mortlock et al., 2007) abolished noncanonical aster formation and delayed cell-like compartment formation (Fig. 3B; observed in six independent experiments), suggesting that Aurora kinase B activity is required for noncanonical aster formation but has limited effect on compartment formation. To further compare the effects of MKLP2 and Aurora kinase B on cell-like compartment formation, we treated the same batch of extracts with their respective inhibitors. We confirmed that microtubule-based cell-like compartments formed in Aurora kinase B-inhibited extracts but not in MKLP2-inhibited extracts (Movie 12).

Next, we inhibited the kinesin Eg5 using the inhibitor *S*-trityl-L-cysteine (STLC). We found that neither the noncanonical aster nor the cell-like compartment formation was affected (Fig. 3C; observed in ten independent experiments). Thus, Eg5 is not required for generating noncanonical asters.

Finally, we tested whether the minus end-directed motor cytoplasmic dynein 1 (referred to here as dynein for simplicity) affects aster formation. We inhibited dynein-mediated transport using the inhibitor p150-CC1 (King et al., 2003; Quintyne et al., 1999). We added purified GST–p150-CC1 to egg extracts and found that noncanonical aster formation was not affected (Fig. 3D; observed in six independent experiments). Formation of cell-like compartments by microtubules was also not significantly affected (Fig. 3E, microtubule panels). However, the ER mostly remained outside the microtubule compartments in dynein-inhibited extracts, whereas in control extracts it accumulated inside the microtubule compartments (Fig. 3E, ER panels and the MT+ER merge panels), suggesting that dynein contributes to the transport of ER into the compartments. We conclude that dynein is not required for generating noncanonical asters.

### Properties of the noncanonical aster

**Fig. 4.**
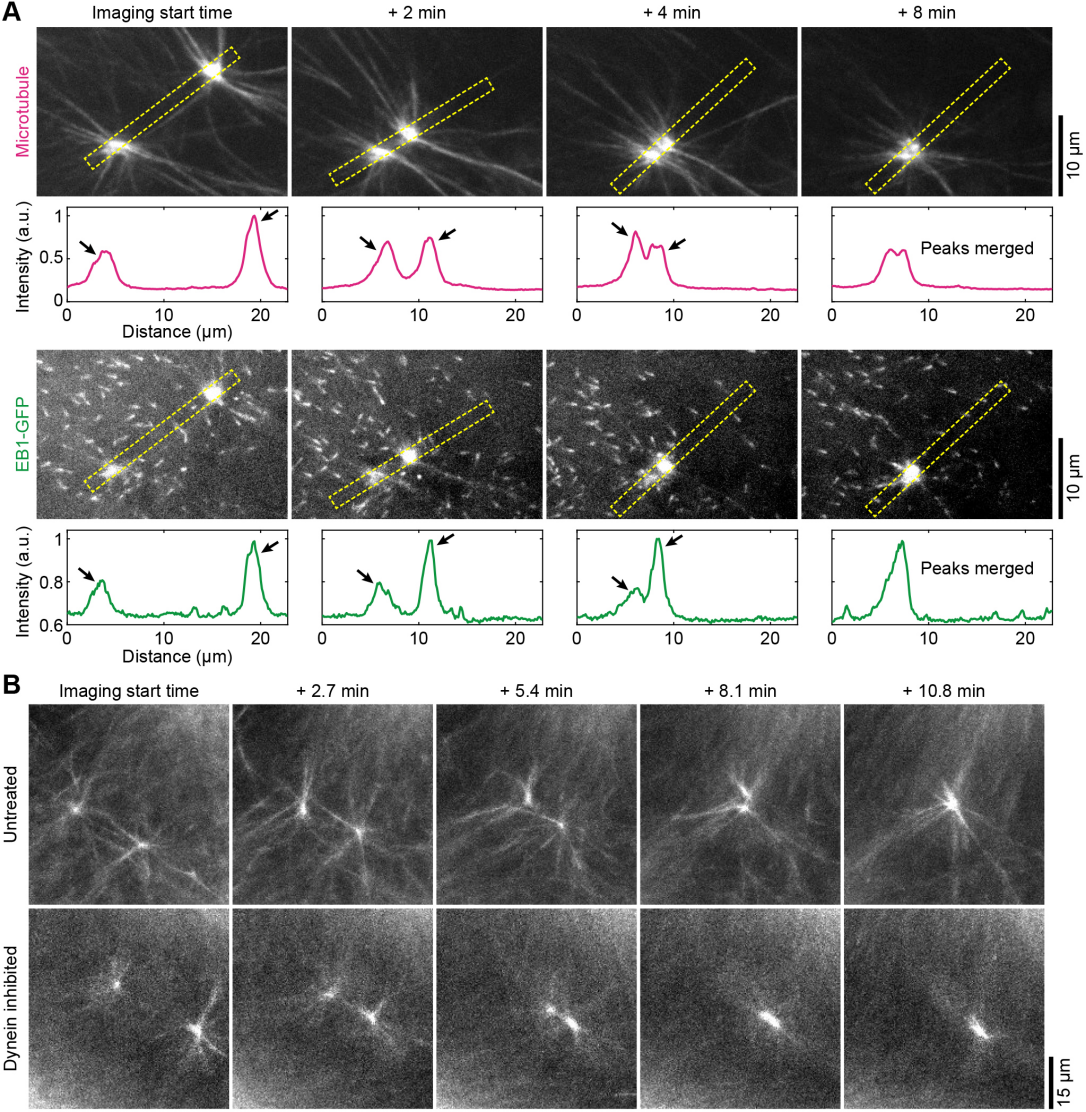
**Noncanonical asters can merge.** (A) Confocal time-lapse montage of microtubule and EB1–GFP dynamics in egg extracts, showing that the centers of two noncanonical asters merged with each another spontaneously, and that the EB1–GFP-enriched regions at the centers also merged. Each image is a maximum-intensity projection of four confocal planes spanning 6 µm of depth. Imaging started at an arbitrary time point after the asters had formed but had not merged. The plot below each image is the fluorescence intensity profile along a 1.65 µm thick, 22.8 µm long line segment (yellow dashed rectangle) that starts at the bottom left and ends at the top right. For each point on the curve in the plot, the horizontal coordinate is the distance from the start of the line segment, and the vertical coordinate is the average fluorescence intensity of the pixels across the width of the line segment at that distance (a.u., arbitrary units). The black arrows indicate intensity peaks for microtubule (second row) and EB1–GFP (fourth row) fluorescence at the aster centers. *n*=9. (B) Confocal images of microtubules in control and GST–p150-CC1-treated extracts, showing that noncanonical asters still merged when dynein was inhibited by 2 µM GST–p150-CC1. *n*=2.

A notable feature of the noncanonical aster was that their centers spontaneously merge, as shown by the microtubule and EB1–GFP dynamics at the aster center (Fig. 4A; Movie 13; observed in 35 cases from nine independent experiments). Fusion of noncanonical asters still occurred when the dynein inhibitor GST–p150-CC1 was added to the extracts (Fig. 4B; observed in 24 cases from two independent experiments), suggesting that dynein is not required.

**Fig. 5.**
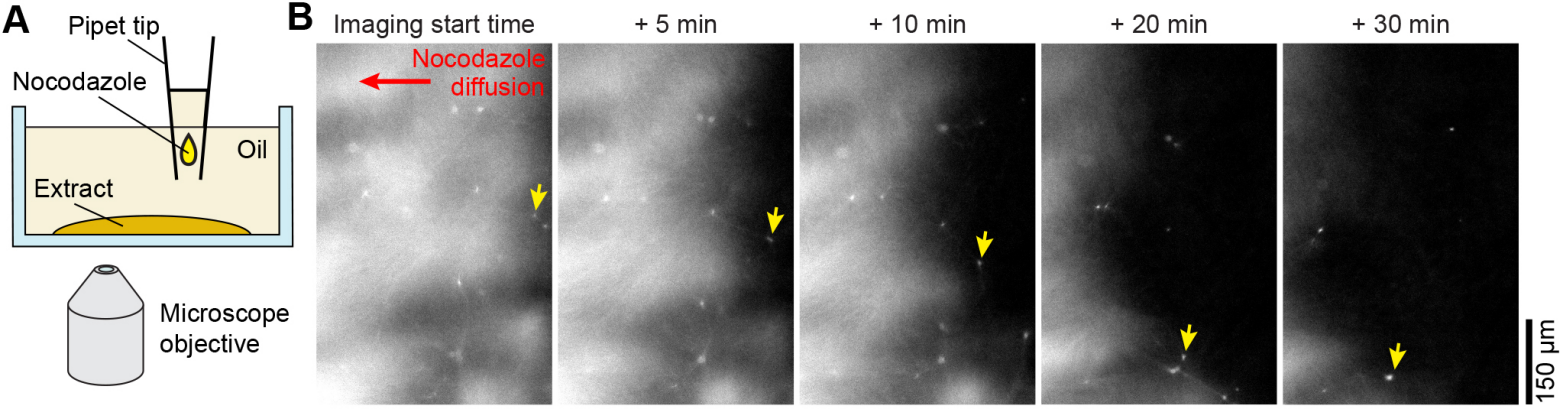
**The center of the noncanonical aster is resistant to microtubule depolymerization.** (A) Experimental setup to supplement extracts with nocodazole after noncanonical aster formation. This setup allowed us to determine whether the noncanonical asters were more resistant to the effect of nocodazole than regular microtubules. (B) Widefield epifluorescence time-lapse images of microtubules in egg extracts with nocodazole administered from the right side of the view. The yellow arrows track the center of an example noncanonical aster that persisted in the presence of nocodazole. Imaging started at an arbitrary time point after the nocodazole droplet had reached the extracts. *n*=3.

The observation that noncanonical asters persist in microtubule-depleted zones raises the possibility that their constituent microtubules are more stable than the surrounding microtubules. To test the idea, we examined the resistance of the aster to microtubule depolymerization. We incubated extracts until noncanonical asters had formed and then treated the extracts with the microtubule depolymerization drug nocodazole (Fig. 5A; see Materials and Methods for technical details). Nocodazole caused microtubules surrounding the aster to depolymerize, but the centers of the noncanonical asters persisted (Fig. 5B; Movie 14; observed in three independent experiments). As the surrounding microtubule network disintegrated, a lattice of noncanonical aster centers coalesced into microtubule foci (Movie 14). These observations suggest that microtubules in the noncanonical aster center are more stable than the surrounding cytoplasmic microtubules.

To further explore the physical properties of the aster, we examined the effect of 1,6-hexanediol, a chemical that interferes with weak hydrophobic protein–protein interactions, on the noncanonical aster. Although 1,6-hexanediol has typically been used at 3.5–10% (mass/volume) (Düster et al., 2021; Kroschwald et al., 2017), we opted for a lower concentration range to minimize potential unintended effects. Egg extracts were supplemented with 1%, 1.5% and 2% 1,6-hexanediol, and microtubule dynamics were tracked by confocal microscopy. Microtubules polymerized in all treated extracts. However, higher doses of 1,6-hexanediol resulted in more severe noncanonical aster defects. At 1% 1,6-hexanediol, vestiges of the aster center and connected astral microtubules were visible (Fig. S5G, yellow arrow and bracket, respectively). In addition, many microtubule bundles at the periphery of the microtubule-depleted zone were still pointing to the much-weakened center in a radial pattern (Fig. S5G, yellow arrowheads). It appeared that this relatively low dose of 1,6-hexanediol preferentially weakened the center in extracts. At 1.5% and 2% 1,6-hexanediol, no aster centers were observed and most of the peripheral microtubule bundles were disorganized (Fig. S5G). Thus, the center of the noncanonical aster is disrupted by 1,6-hexanediol (consistent across 22 samples from four independent experiments). We conclude that non-specific weak hydrophobic interactions contribute to noncanonical aster organization.

We next sought to determine how 1,6-hexanediol affects cell-like compartment formation. We treated extracts with 2% 1,6-hexanediol and monitored microtubule dynamics using epifluorescence microscopy. Microtubules formed in a spatially uniform manner in both 2% 1,6-hexanediol-treated and untreated control extracts (Fig. S5H, 3–28 min; Movie 15). Quantification of microtubule fluorescence revealed rapid polymerization dynamics during this initial period (Fig. S5I; consistent across three independent experiments). Microtubule-depleted zones appeared in the treated extracts, but with a temporal delay and in much lower numbers compared to the control (Fig. S5H, 51 min; Movie 15). Normal-looking cell-like compartments did not form in 2% 1,6-hexanediol-treated extracts, although some partitioning of microtubules did occur after extended incubation (Fig. S5H, 243 min; Movie 15). These patterning dynamics were consistent across seven samples from three independent experiments. These results show that perturbing the extract with 1,6-hexanediol disrupts cell-like compartment assembly.

### The noncanonical aster spatially organizes cytokinesis components

We next explored the potential biological functions of the noncanonical aster. A few observations suggest that they might be linked to cytokinesis. First, the asters arise in the interphase of the cell cycle, which is when cytokinesis normally occurs in early *Xenopus* embryonic cells (Nguyen et al., 2014; Wühr et al., 2010). Second, the asters localize to a spatial domain analogous to the cytokinesis plane. In a dividing early *Xenopus* embryonic cell, the cytoplasm assembles two wreath-like microtubule arrays separated by a microtubule-depleted border before the cytokinesis furrow forms at that border (Mitchison and Field, 2021; Mitchison et al., 2012; Wühr et al., 2010). This border accumulates cytokinesis signals and is thought to specify the cytokinesis plane (Nguyen et al., 2014; Wühr et al., 2010). The same phenomenon is also observed in zebrafish (Rinaldin et al., 2024 preprint). In *Xenopus* egg extracts, a similar architecture arises since the cell-like compartments each also contain a wreath-like microtubule array, and they are also separated by microtubule-depleted borders where noncanonical asters were localized (Fig. 1E). We thus examined whether noncanonical asters interact with components of the cytokinesis pathway.

**Fig. 6.**
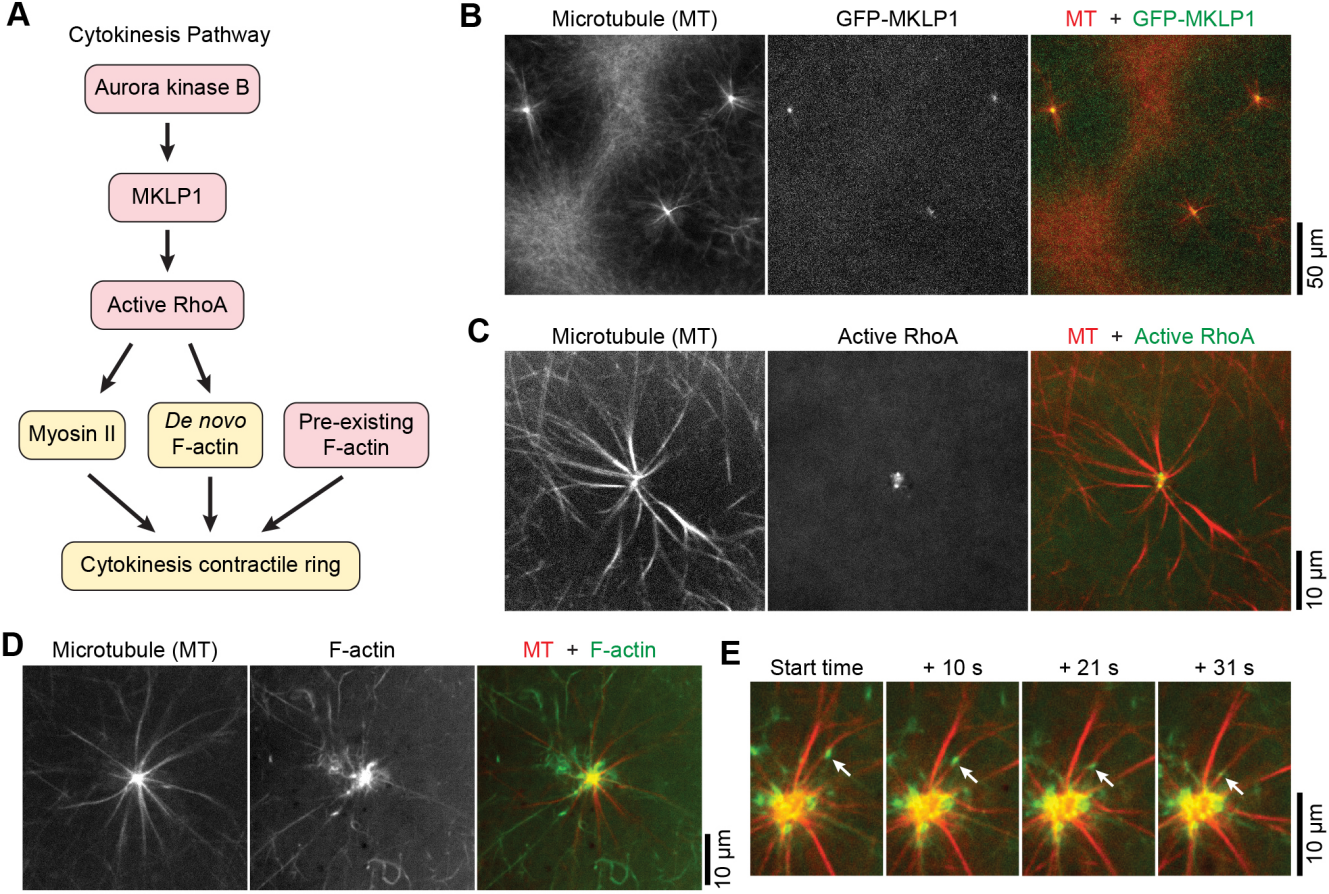
**The noncanonical aster spatially organizes cytokinesis components.** (A) A diagram of the cytokinesis pathway. The four components investigated in the present study are marked in pink. *De novo* F-actin refers to newly polymerized F-actin due to RhoA activity. (B) Confocal images of microtubules and added GFP–MKLP1 in egg extracts, showing that GFP–MKLP1 localized to the centers of noncanonical asters. *n*=6. (C) Single-plane confocal images of microtubules and active RhoA in egg extracts. Active RhoA is visualized using GST–GFP–rGBD. *n*=7. (D) Single-plane confocal images of microtubules and F-actin in a noncanonical aster. F-actin is visualized using LifeAct–GFP. *n*=7. (E) Time-lapse montage of single-plane confocal images of microtubules (red) and F-actin (green) in a noncanonical aster. The montage shows the movement of an F-actin punctum (tracked by the white arrows) along an astral microtubule bundle. Imaging started at an arbitrary time point after the aster had formed. *n*=2.

Fig. 6A briefly summarizes the cytokinesis pathway (Alberts et al., 2022; Basant and Glotzer, 2018). We have shown earlier that Aurora kinase B, a cytokinesis-related protein, is required for noncanonical aster formation (Fig. 3B). To test whether the noncanonical aster interacts with other components of the cytokinesis pathway, we examined the localization of MKLP1, a downstream target of Aurora kinase B. We added GFP–MKLP1 to extracts and found that it localized to the center of the noncanonical aster (Fig. 6B; observed in six independent experiments). Next, we examined the localization of active RhoA, a cytokinesis component downstream of MKLP1 (Fig. 6A). To visualize active RhoA, we supplemented the extracts with purified recombinant fluorescent protein GST–GFP–rGBD, which binds specifically to active RhoA (Benink and Bement, 2005). We found that active RhoA also localized to the center of the noncanonical aster, as indicated by the GST–GFP–rGBD fluorescence (Fig. 6C; Fig. S6A). This localization pattern persisted in noncanonical asters even after cell-like compartments had formed (Fig. S6B,C). No enrichment of active RhoA was observed further away from the aster center within the aster territory (Fig. S6C). This active RhoA localization pattern was consistent in 23 noncanonical asters from seven independent experiments.

To further explore the link between the noncanonical aster and the cytokinesis pathway, F-actin dynamics were examined. F-actin is an essential component of the cytokinesis contractile ring in cells (Miller, 2011). Even though locally polymerized F-actin stimulated by active RhoA (referred to as *de novo* F-actin in Fig. 6A) contributes to the contractile ring, pre-existing F-actin shipped from distal locations is thought to be preferentially used for ring assembly (Cao and Wang, 1990). Because the localization of the noncanonical aster at the cell-like compartment borders is reminiscent of the cytokinesis site in cells, we asked whether pre-existing F-actin filaments are recruited to the aster like they are recruited to the cytokinesis site in cells. Purified LifeAct–GFP protein was added to the extracts to visualize F-actin (Belyy et al., 2020; Riedl et al., 2010) and imaged together with microtubules using confocal microscopy. We found that F-actin was enriched at the center of the noncanonical aster (Fig. 6D; observed in seven independent experiments). Distal F-actin continuously moved towards the aster center (Movie 16). In some cases, F-actin puncta could be seen moving along astral microtubule bundles towards the center (Fig. 6E; observed in four samples from two independent experiments), suggesting microtubule motor-based transport.

Together, our data link the noncanonical aster to five components of the cytokinesis pathway: MKLP2, Aurora kinase B, MKLP1, active RhoA and F-actin (Fig. 3A,B, Fig. 6B–D). In particular, the center of the aster spatially focuses MKLP1, active RhoA, and F-actin.

## DISCUSSION

In this study, we characterize centrosome-independent asters that self-organize in *Xenopus* egg extracts (Fig. 1). We refer to these asters as noncanonical asters since they form in the apparent absence of the classic microtubule aster organizer centrosome. We show that most growing microtubules around the noncanonical aster center orient their plus ends toward the center, in contrast to those in the centrosome aster (Fig. 2). The aster center is a microtubule plus end hub, as it accumulates EB1–GFP, a plus end-binding protein, and kinesin-1–GFP, a plus end-tracking motor (Fig. 2). Formation of the noncanonical aster requires the microtubule motor MKLP2 and Aurora kinase B activity (Fig. 3). The centers of the aster can fuse in a dynein-independent manner (Fig. 4) and are stable against microtubule depolymerization (Fig. 5). Finally, we show that the aster center accumulates cytokinesis components including MKLP1, active RhoA and F-actin (Fig. 6).

Noncanonical asters are similar to the antiparallel microtubule bundles found in the cell division plane (Basant and Glotzer, 2018; Field et al., 2019; Nguyen et al., 2014, 2018; Verma et al., 2019). Both structures are MKLP2-dependent microtubule plus end hubs that localize to the border of cell-like compartments (Fig. 1E; Fig. 3A) and recruit the cytokinesis components MKLP1, active RhoA and F-actin (Fig. 6B–D) (Nguyen et al., 2014, 2018). They both clear nearby microtubules while allowing directly connected microtubules to persist (Fig. 1D; Fig. 2F) (Field et al., 2019; Nguyen et al., 2014). The main difference between these two structures is that noncanonical asters are radial arrays, whereas the bundles are linear parallel arrays. We therefore postulate that the noncanonical aster is a variant form of the antiparallel microtubule bundles in the spindle midzone and aster–aster interface in early embryonic cells (Nguyen et al., 2014, 2018), exhibiting a different morphology because they form without centrosomes and chromatin.

The properties of MKLP2 and Aurora kinase B, two key factors required for noncanonical aster formation (Fig. 3A,B), suggest potential mechanisms underlying this process. As a plus end-directed motor, MKLP2 can potentially organize microtubules into a polarity-inverted aster by focusing their plus ends. In support of this notion, plus end motors have been shown to organize microtubules into plus-end-at-center asters *in vitro* (Nédélec et al., 2001; Nitta et al., 2021; Surrey et al., 2001; Urrutia et al., 1991). Aurora kinase B could be transported by MKLP2 (Adriaans et al., 2020; Gruneberg et al., 2004) to the plus end aster center, potentially stabilizing it and clearing the surrounding microtubules. In support of these roles, Aurora kinase B has been shown to promote microtubule stability and antiparallel plus end bundle formation (Field et al., 2015; Nunes Bastos et al., 2013). Furthermore, Aurora kinase B-coated beads clear most surrounding microtubules but retain radial arrays of thick microtubule cables directly connected to them, generating microtubule patterns that resemble noncanonical asters (Field et al., 2019). Therefore, one potential mechanism for noncanonical aster formation comprises MKLP2-mediated microtubule plus end focusing and Aurora kinase B-dependent microtubule clearance. The initial uniform microtubule polymerization likely increases microtubule density enough for MKLP2 to crosslink them. This model is consistent with the experimentally observed noncanonical aster formation dynamics. Alternatively, other plus end-directed motors might focus microtubules into an aster, while MKLP2 primarily contributes to the microtubule-depleted zone by transporting Aurora kinase B to the aster center. Candidates for such aster-generating motors include kinesin-1 and MKLP1, since both are expressed in the *Xenopus* egg (Wühr et al., 2014) and localize to the center of the noncanonical aster (Fig. 2H; Fig. 6B). Finally, other microtubule-associated proteins, such as the plus end-bundling protein Prc1 and the motor Kif4A, might also play a role in aster formation (Nguyen et al., 2014, 2018).

Cell-like compartment formation does not require noncanonical asters, as Aurora kinase B inhibition abolishes asters but not the compartments (Fig. 3B). However, the two processes might share certain upstream regulators. For example, MKLP2 is required for both the asters and compartments (Fig. 3A; Movie 12). In addition, the different phenotypes from Aurora kinase B and MKLP2 inhibition suggest that MKLP2 might play a role other than transporting Aurora kinase B in compartment formation. Although compartment formation appears more efficient when noncanonical asters are unperturbed, it is difficult to discern whether the efficiency loss is caused by the loss of asters or the perturbations themselves. Aurora kinase B inhibition by barasertib abolishes the aster, and the cell-like compartments form with a substantial delay (Fig. 3B). Treatment with 2% 1,6-hexanediol abolishes the aster, and in the treated extracts cell-like compartment formation is defective (Fig. S5H). However, it is possible that the defect and delay are due to non-specific effects of 1,6-hexanediol, functions of Aurora kinase B unrelated to the noncanonical aster, or the off-target effects of barasertib. For instance, 1,6-hexanediol above 0.5% (mass/volume) may impair certain kinases and phosphatases (Düster et al., 2021). Therefore, our perturbation experiments do not establish a causal link between aster formation and compartment formation.

Extract variability and confocal imaging *z*-range might lead to variations in the observed shapes of microtubule-depleted zones in the noncanonical asters. For example, the depleted zones in Fig. 1B are circular, but those in Fig. 3B appear more like a depleted band. The band-like appearance of the latter is mainly because multiple circular depleted zones within the captured *z*-range emerge close to each other and thus appear interconnected. In addition, the asters within these circular zones are out of the captured *z*-range in some initial frames, leading to the appearance of depleted bands without asters inside. About 8% of our images fall into this category, based on data from 25 independent experiments.

The interphase extracts used for noncanonical aster assembly lacked nuclear compartments because no sperm nuclei were added. Consequently, factors normally sequestered in the nucleus were present in the cytoplasm, making the extracts nucleocytoplasmic. Some of those factors might regulate self-organization by influencing microtubule motor activity. One such factor, the nuclear mitotic apparatus protein (NuMA, also known as NUMA1), has recently been shown to regulate dynein (Colombo et al., 2025) and thus might promote cell-like compartment formation by focusing microtubule minus ends. However, inhibiting dynein transport with GST–p150-CC1 does not have significant effects on the radial organization of microtubules in cell-like compartments (Fig. 3E). Therefore, the NuMA–dynein axis does not appear to be required for this process. Nevertheless, it would be interesting to further explore the effects of other nuclear factors on noncanonical aster and cell-like compartment formation.

The mitotic spindle spatially organizes cytokinesis signals to position the cleavage furrow in animal cells. These signals are focused at the cell equator by the spindle body and centrosome asters (Basant and Glotzer, 2018; Verma et al., 2019). Previous studies show that spatially focused cytokinesis signals can be generated by centrosome asters alone (Field et al., 2015; Nguyen et al., 2014). The present work shows that spatially focused cytokinesis signals can arise in the absence of both the spindle body and centrosome asters, underscoring the self-organizing capacity of the cytoskeleton and the robustness of the cytokinesis program.

## MATERIALS AND METHODS

### *Xenopus laevis* egg extract preparation

All *Xenopus* experiments and animal care followed protocols (#21338) approved by the Institutional Animal Care and Use Committee (IACUC) of the University of Southern California. *Xenopus laevis* frogs were obtained from Xenopus 1. Interphase-arrested cytoplasmic extracts were prepared as described previously (Cheng and Ferrell, 2021), with the modification that the F-actin inhibitor cytochalasin B was not added to the extracts to allow actin polymerization. Previously established egg quality criteria (Cheng and Ferrell, 2021) were followed, and only high-quality eggs were used for *de novo* assembly experiments. If the extracts appeared turbid, they were clarified by centrifuging for an additional 10 min at the same speed at which they were originally prepared (Murray, 1991). Fresh extracts were kept on ice and used within 4 h. Demembranated sperm nuclei were prepared as described previously (Deming and Kornbluth, 2006) and were added into the extracts at a final concentration of ∼8 nuclei/µl.

### Plasmids and cloning

EB1–GFP was from the plasmid pET21a-EB1-GFP, which was a gift from Dr Yixian Zheng in the Department of Embryology, Carnegie Institution for Science, Baltimore, MD, USA. The construct contained a 6×His tag at the C terminus of the EB1–GFP for affinity protein purification. Kinesin-1–GFP was from the plasmid pET17_K560_GFP_His (Addgene plasmid 15219; http://n2t.net/addgene:15219; RRID: Addgene_15219; deposited by Ron Vale). The construct contained a 6×His tag at the C terminus of the kinesin-1–GFP sequence for affinity protein purification. LifeAct–GFP was originally in the plasmid pLenti.PGK.LifeAct-GFP.W, kindly provided by Dr Rusty Lansford at the Keck School of Medicine, University of Southern California, USA. The LifeAct–GFP sequence was subsequently cloned into the pET21a vector derived from the plasmid pET21a-EB1-GFP (see above) using Gibson Assembly for protein expression. The construct contained a 6×His tag at the C terminus of the LifeAct–GFP sequence for affinity protein purification. The GFP–rGBD sequence was from the plasmid GFP–rGBD (Addgene plasmid 26732; http://n2t.net/addgene:26732; RRID:Addgene_26732; deposited by William Bement). The GFP–rGBD sequence was subsequently cloned into the pGEX-4T-1 vector derived from a GST-GFP-NLS plasmid from a previous study (Cheng and Ferrell, 2019) using Gibson Assembly to create pGEX-GFP-rGBD for protein expression. The construct contained a glutathione S-transferase (GST) tag at the N terminus of GFP–rGBD for affinity protein purification. The GFP–MKLP1 was from the plasmid pCS2-GFP-MKLP1 (Addgene plasmid 140566; http://n2t.net/addgene:140566; RRID:Addgene_140566; deposited by Heidi Hehnly). This construct includes an SP6 promoter, allowing for *in vitro* transcription and translation using wheat germ extract-based protein expression systems. The chicken DCTN1 p150Glued AA 217–548 (CC1) was from the plasmid pVEX-CC1 (Addgene plasmid 74170; http://n2t.net/addgene:74170; RRID:Addgene_74170; deposited by Trina Schroer). The CC1 sequence was subsequently cloned into pGEX expression vector, with an N terminal GST tag added to facilitate affinity purification of GST–p150-CC1.

For LifeAct–GFP, GST–GFP–rGBD and GST–p150-CC1, which required backbone switching, the DNA fragments of the functional inserts were amplified with Phusion High Fidelity DNA polymerase (M0530S, New England BioLabs). Primers were synthesized by Integrated DNA Technologies (IDT). PCR reactions were verified by gel electrophoresis, and PCR products were purified using the QIAquick PCR Purification Kit (28106, Qiagen). Plasmid construction was carried out using Gibson Assembly Master Mix (E2611, NEB). The ligated products were transformed into chemically competent *E. coli* DH5α cells (C2987H, NEB), and transformants were selected on Luria-Bertani (LB) agar plates containing 50 µg/ml ampicillin. Positive clones were confirmed by QIAprep Spin Miniprep Kit (27106, Qiagen) and DNA sequencing (Retrogen Inc. for sanger sequencing and Plasmidsaurus for whole-plasmid sequencing).

### Protein expression and purification

Plasmids carrying the protein expression constructs were transformed into *E. coli* BL21(DE3) cells (C2527I, NEB) and cultured on appropriate antibiotic selection plates. A single colony was used to inoculate 3 ml of LB medium containing 50 µg/ml ampicillin, and the culture was grown at 37°C with shaking at 200 r.p.m. until the optical density at 600 nm (OD600) reached 0.5–1. The 3 ml culture was then diluted into 500 ml of fresh LB medium containing 50 µg/ml ampicillin and grown at 16°C with 0.15 mM isopropyl β-D-1-thiogalactopyranoside (IPTG; BP1755-1, Fisher Scientific) for induction overnight with shaking at 200 r.p.m. Overnight-grown *E. coli* cells were harvested by centrifugation at 6000 ***g*** for 15 min at 4°C.

For expressing EB1–GFP, kinesin-1–GFP and LifeAct–GFP, which had a 6×His tag, the harvested cell pellet was resuspended in 40 ml lysis buffer [2 mM imidazole, 50 mM Tris-HCl pH 7.9, 200 mM NaCl, 1% glycerol, 4 mM MgSO_4_, 1 mM dithiothreitol (DTT), 1 mg/ml lysozyme (89833, Thermo Fisher)] with EDTA-free protease inhibitor cocktail tablets (A32955, Thermo Fisher) and Benzonase nuclease (E1014-25KU, Sigma), and incubated on ice for 30 min. Cells were lysed by sonication on ice for 6 min. The lysate was clarified by centrifugation at 17,000 ***g*** for 45 min at 4°C, and the supernatant was filtered through a 0.22 µm filter. The supernatant was loaded onto a column loaded with Ni-NTA affinity beads (30210, Qiagen) pre-equilibrated with lysis buffer. The column was washed twice with wash buffer (20 mM Tris-HCl, 200 mM NaCl, 5 mM imidazole) to remove non-specifically bound proteins. The His-tagged protein was eluted with elution buffer (20 mM Tris-HCl pH 7.5, 200 mM NaCl, 200 mM imidazole).

For expressing GST–GFP–rGBD and GST–p150-CC1, which had GST tags, the harvested cell pellet was resuspended in 40 ml lysis buffer (50 mM Tris-HCl pH 7.9, 200 mM NaCl, 1% glycerol, 4 mM MgSO_4_, 1 mM DTT, 1 mg/ml lysozyme) with EDTA-free protease inhibitor cocktail tablets (A32955, Thermo Fisher) and Benzonase nuclease (E1014-25KU, Sigma), and incubated on ice for 30 min. Cells were lysed by sonication on ice for 6 min. The lysate was clarified by centrifugation at 17,000 ***g*** for 45 min at 4°C, and the supernatant was filtered through a 0.22 µm filter. The supernatant was loaded onto a column loaded with glutathione agarose beads (16100, Thermo Fisher) pre-equilibrated with lysis buffer. The column was washed twice with wash buffer (20 mM Tris-HCl, 200 mM NaCl) to remove non-specifically bound proteins. The GST-tagged protein was eluted with elution buffer (1 M HEPES pH 7.0, 5 M NaCl, 1 M DTT, and reduced glutathione; pH adjusted to 7.8).

The buffer of the eluted fractions containing the His-tagged or GST-tagged protein was exchanged to storage buffer (20 mM HEPES, 150 mM KCl, pH 7.7) using a PD-10 desalting column (95017-001, VWR), and the protein was concentrated using a Pierce protein concentrator (88517, Thermo Fisher) with a 10 kDa molecular weight cutoff.

Protein concentration was determined using the Pierce BCA Protein Assay Kit (23225, Thermo Fisher) with bovine serum albumin (BSA) as a standard. Protein purity was assessed by SDS-PAGE, and gels were stained with Coomassie Brilliant Blue (1610400, Bio-Rad).

GFP–MKLP1 was expressed using the TnT SP6 High-Yield Wheat Germ Protein Expression System (L3261, Promega), following the manufacturer's protocol for *in vitro* protein synthesis. After the *in vitro* protein synthesis reaction was completed, the reaction mix was added to extracts at 1:30 (v/v).

### Chemicals and fluorescently labeled tubulin

All chemicals used in the study were from Millipore-Sigma unless otherwise noted. *S*-trityl-L-cysteine (L14384.03, Thermo Fisher) was added to extracts to a final concentration of 100 µM. Paprotrain (HY-101298, MedChemExpress) was added to extracts at various concentrations as indicated, including 50 µM and 100 µM. For Aurora kinase B inhibition experiments, barasertib-HQPA (HY-10126, MedChemExpress) was added to a final concentration of 40 µM. For 1,6-hexanediol experiments, Solid 1,6-hexanediol was dissolved in UltraPure distilled water (10977023, Thermo Fisher) to form an 80% (weight/volume) stock solution. This stock solution was added to extracts to reach final concentrations of 1%, 1.5% and 2% 1,6-hexanediol (w/v). HiLyte 488-labeled tubulin (TL488M-A, Cytoskeleton, Inc.) or HiLyte 647-labeled tubulin (TL670M-A, Cytoskeleton, Inc.) were added to the extracts to a final concentration of 1 µM. For dynein inhibition, purified GST–p150-CC1 was added to a final concentration of 0.68 µM or 2 µM as indicated in the text.

### Nocodazole administration

An extract droplet with a volume of 2.4 µl was deposited on the bottom of a well in a plastic 96-well plate (351172, Corning) and covered on top with mineral oil (330760, Sigma). The extract droplet was incubated and imaged until noncanonical asters started to form. Nocodazole originally dissolved in dimethyl sulfoxide (DMSO) at 24 mM was diluted in water to a hazy working solution of 2.4 mM. The working solution was loaded into a glass microinjection needle and dispensed into mineral oil as 40 nl droplets with a microinjector (MPPI-3, Applied scientific instrumentation). A single 40 nl nocodazole droplet together with oil was picked up by a regular hand pipet with a 10 µl tip. The tip contained the nocodazole droplet suspended in oil. The 40 nl nocodazole droplet was then dispensed onto the extract droplet under oil using the hand pipet. The nocodazole droplet descended under gravity, eventually fused with the extract droplet, and diffused from the initial impact site, as confirmed by live imaging. After thorough diffusive mixing the final concentration of nocodazole in the extract was 40 µM.

### Image acquisition and imaging probes

Extracts were thoroughly mixed upon the addition of reagents. Extracts were placed in a sealed imaging chamber formed by sandwiching an imaging spacer with a glass coverslip and a glass slide (see schematics in Fig. S1A). The surfaces in contact with the extract were coated with Aquapel (PGW Auto Glass LLC, Cranberry Twp, PA, USA) or lined with fluorinated ethylene propylene (FEP) tape (Cheng and Ferrell, 2021) (23-FEP-2-36, CS Hyde Company). The imaging spacers were double-sided sticky tapes ranging from ∼0.06 mm to 0.12 mm in height. The thinner spacers were made by punching a hole in a double-sided sticky tape (B09F9LMP3H, Amazon) with a single-hole punch (item 825232, Office Depot). The 0.12 mm spacers were from Grace Bio-Labs (654002). Imaging commenced within 1–3 min after extracts were mounted into the imaging chamber. In any figure that displays a time-lapse image montage, if absolute time labels are used (e.g. 1 min, 2 min, etc.), then time point zero is the time when extracts start room temperature incubation. If relative time labels are used (e.g. +1 min, +2 min, etc.), then imaging starts at a time point specified in the figure legend.

Confocal images were collected with a Photometrics Prime BSI Express sCMOS camera connected to a Yokogawa CSU-X1 spinning disk confocal module and a Nikon Ti2-E inverted microscope with a 20× Nikon objective (NA 0.8) and a 60× Nikon objective (NA 1.42). For single-plane confocal images, the plane (optical section) thickness was ∼1.3 µm. Epifluorescence images were acquired with a Leica K8 sCMOS camera connected to a Leica DMi8 inverted microscope with a 5× Leica objective (NA 0.15). Imaging was carried out at room temperature.

SiR-tubulin (CY-SC002, Cytoskeleton, Inc.) was used to visualize microtubules unless otherwise noted. SiR-tubulin was added to extracts to a final concentration of 300 nM. ER-Tracker Red (E34250, Thermo Fisher) was added to 1 µM to visualize the ER. LifeAct–GFP was added to final concentrations of 0.5–1 µM to extracts to visualize F-actin. EB1–GFP was added to 150 nM. Human kinesin-1–GFP was added to 100 nM. GST–GFP–rGBD was added to 300 nM.

When using EB1–GFP to determine microtubule orientation, purified EB1–GFP protein and rhodamine-labeled tubulin (TL590M-B, Cytoskeleton, Inc.) were added to egg extracts to visualize EB1 and microtubules, respectively (Fig. 2A). Demembranated *Xenopus* sperm nuclei, which contained centrosomes, were added to egg extracts to generate centrosome asters. SiR-tubulin and purified EB1–GFP were added to visualize microtubules and their growing plus ends (Fig. 2C).

### Image processing and quantification

Fluorescence intensity time series plots were generated as follows. For Fig. 1C, Fig. S1C and Fig. S5I, the fluorescence intensity data were calculated using the complete sequences of time-lapse images from which the representative images shown in Fig. 1B, Fig. S1B and Fig. S5H were selected. Note that Fig. 1B and Movie 2 show maximum-intensity projection images for visual clarity, whereas Fig. 1C uses the sum-of-slices intensity projection image at each time point for quantification to more accurately capture the total fluorescence across all *z*-planes at that time point. The confocal *z*-slices are raw images that are not flat-field corrected to preserve the fidelity of the intensity values. As a result, the uneven illumination of the field intrinsic to microscope optics, which makes the edges and corners of the images appear darker, is readily noticeable in Fig. 1B and Movie 2. The raw total intensity of an image at a given time point was the sum of all pixel intensities in that image. The intensity was calculated for each image in the time-lapse sequence. The resulting time series intensity data were subsequently normalized by the maximum intensity among all data points being (co-)plotted. In the case where two time series were co-plotted on the same axes, the intensity value used to normalize the data was the common maximum intensity among both time series. Intensity values and units across different experiments and plots are not comparable.

The angle metric *θ* that describes microtubule orientation relative to a designated reference center point was calculated from EB1–GFP images through the following procedure. (1) We manually identify from the image of each comet the front extremity point at the comet head (henceforth ‘comet front tip’) and the rear extremity point at the comet tail (henceforth ‘comet rear tip’) and extract the coordinates of those two points. We define the vector that starts at ‘comet rear tip’ and ends at ‘comet front tip’ as the comet vector (Fig. 2B; Fig. S3A,B). (2) To implement a consistent distance scope, we calculate the angle metric *θ* for only the comets whose ‘comet front tips’ fall into the bounds of a circular region with a 22 µm radius (Fig. 2B). (3) We determine the reference center point from the EB1–GFP image. For images containing asters, the aster center enriched in EB1–GFP is manually segmented and represented by a polygon (Fig. S3C). The geometric center of this polygon is determined using the MATLAB function ‘centroid’ and is designated as the reference center point (Fig. S3C, five-pointed stars). For images with no asters, the reference center point is arbitrarily chosen in the middle of the image to allow a circular region with a 22 µm radius from the center point to fall within the bounds of the image. (4) We define the vector that begins at the reference center point and ends at the ‘comet front tip’ as the radial vector. The coordinates for the reference center point and the ‘comet front tip’ are extracted from images. (5) The angle *θ* is defined as the angle between the comet vector and the radial vector. Because the coordinates of both vectors have been extracted from images, angle *θ* is calculated by applying the law of cosines to the two vectors. Accordingly, the range of the value of *θ* was [0, 180°]. A microtubule aster is approximately radially symmetric in three dimensions, so a single confocal plane that crosses its geometric center should represent all other planes that also cross the center, thereby representing the entire three-dimensional volume. Therefore, a still image at a single time point for such a single confocal plane was used for the calculations. If *θ*>90°, the microtubule plus end points towards the center, and the microtubule (and EB1–GFP comet) is centripetal or ‘incoming’. If *θ*≤90°, it points away from the center and the microtubule is centrifugal or ‘outgoing’ (Fig. 2B; Fig. S3B). In calculating the percentage of EB1–GFP comets pointing toward or away from the center using *θ* values, only the comets near the center point (within a 22 µm radius) were used. To assess microtubule orientation in the no-aster scenario, we chose an arbitrary point in the middle of the image as the center and analyzed EB1–GFP comets within a 22 µm radius (Fig. 2C, circled region R3).

To track EB1–GFP comet movement, we used the software package u-track (version 2.2), which included the microtubule plus end-tracking functionality of plusTipTracker (Applegate et al., 2011). The EB1–GFP track data were exported from the package in MATLAB and the average direction for each track was calculated by averaging the angles of all segments of the track (a track segment is the vector pointing from the comet position in one frame to the comet position in the next frame). A color disk using the periodic HSV color map in MATLAB was generated to map a unique color to each direction pointing from the center of the disk to the edge of the disk. The average direction of each EB1–GFP track was color coded using this color disk.

To determine the numbers of EB1–GFP comets that (1) go into the noncanonical aster center or vanish within a 10 µm radius from the center, and (2) go out from the center, we chose a single confocal *z*-plane containing the aster center and manually counted the comets by visual inspection of EB1–GFP movies from different experiments. We counted the EB1–GFP comets over a ∼3 min time window.

To visualize kinesin-1–GFP movement around the noncanonical aster in Movie 11, we used BaSiC tool (Schindelin et al., 2012) in the Fiji software plug-in to correct for uneven illumination and temporal signal decline due to photobleaching (temporal drift), with all parameters left at default values.

To calculate the fractions of total tubulin fluorescence inside and outside the cell-like compartments, we manually segmented cell-like compartments from fluorescence images of labeled tubulin in extracts. The fraction of signals inside the compartments was the sum of pixel intensities within all compartments divided by the sum of all pixel intensities in the image. The fraction of signals outside the compartments was 1 minus the fraction of the signals inside compartments. An example of compartment segmentation is shown in Fig. S3.

For the analysis of EB1–GFP and SiR-tubulin fluorescence in Fig. 4A (acquired with a 60× oil immersion objective) and Fig. S4A (acquired with a 20× air objective), each plot beneath an image is an intensity profile along a thick scan line generated as follows. First, the image was imported into the Fiji software (Schindelin et al., 2012) and a 15-pixel-wide line segment (henceforth referred to as a ‘scan line’) was drawn directionally from bottom left to top right that covered the features of interest (e.g. the centers of the two noncanonical asters in Fig. 4A) using the line tool in Fiji. Scan lines for each time point were placed at identical positions in the microtubule and EB1–GFP images corresponding to that time point. Due to the substantial width of the scan line, it is indicated using a rectangular box with yellow dashed lines in the figures. After the scan line was drawn on top of the image in the Fiji software, we selected ‘Analysis’ and then ‘Plot profile’ to generate the profile plot. The horizontal axis of the profile plot is the distance along the scan line counting from the start of the line (the bottom left end). The vertical axis is the raw intensity values, which must be corrected for temporal drift (caused by factors such as photobleaching) for each time point so that the intensity values in the profiles from different time points are comparable. For the same experiment, the scan lines at different time points were drawn with the same length using the Fiji ‘makeLine’ function in the macro tool dialogue box, so the horizontal axes of the profile plots across all time points always had the same distance range. However, the scan lines for different time points did not necessarily have the same position and orientation, because the features of interest moved around over time, and the scan line must be adjusted accordingly to capture them. Next, the raw intensity profile data were exported from Fiji and corrected for temporal drift. The baseline fluorescence subject to temporal drift was estimated using the BaSiC tool (Peng et al., 2017). The maximum-intensity projection images (for confocal data) or raw images (for epifluorescence data) were supplied to the BaSiC tool (as a Fiji software plug-in) as the input data. Specifically, we imported the image data into Fiji and chose the menu options ‘Plugins’ followed by ‘BaSiC’. In the BaSiC plug-in interface, we chose the option ‘Estimate both flat-field and dark-field’ in the ‘Shading_model’ section, and the option ‘Replace with temporal mean’ in the ‘Temporal_drift of baseline’ section to generate the temporal drift correction curve in the output. All other parameters in the BaSiC plug-in interface were left at default values. In the output, the baseline data were accessed through either the ‘Basefluor’ window or the ‘Temporal components’ window. The data comprised a list of intensity values, each corresponding to an input image in the time series. To correct the raw line scan profiles at a given time point for temporal drift, we subtracted the estimated baseline intensity value at that time point from the raw profile values (for rationale, see the model and equation 2 in the BaSiC study; Peng et al., 2017). The temporal drift-corrected intensity values were then normalized by the maximum intensity among the time points selected for plotting. Therefore, the final corrected profile plots for a specific fluorescence channel share the same intensity range and units (arbitrary units, a.u.) and are directly comparable. Because the excitation wavelength, light source power and fluorescence efficiency differ for different fluorophores, the normalized intensity units and intensity values across different fluorophores are not directly comparable.

The images in Fig. 3B were from larger stitched images obtained as follows. A tiled array of images spanning a large field of view were acquired. Each image tile was flat-field corrected using the BaSiC tool (Peng et al., 2017), which was available as a Fiji/ImageJ plug-in. The parameters for the BaSiC tool were set as follows. Both dark-field and flat-field shading were estimated and used for correction. All other parameters were left at default values. The image tiles from each time point and each confocal plane were stitched together by a custom MATLAB script to generate an output image with a large field of view. Stitched large images from nine confocal *z*-planes were used to generate maximum-intensity projection images using Fiji/ImageJ. Regions of interest from the projection images were then selected for display in the figure. All other images in the study were original raw images not corrected for uneven illumination.

## Supplementary Material

10.1242/joces.263766_sup1 Supplementary information
