# Supplementary material for: An acentrosomal aster with atypical microtubule polarity recruits cytokinesis signals to its center in Xenopus egg extracts
Source: J Cell Sci. 2025 Sep 26;138(18):jcs263766. doi: 10.1242/jcs.263766 (PMC12516129; doi:10.1242/jcs.263766)
Supplement: Supplementary information [file joces-138-263766-s1.pdf]

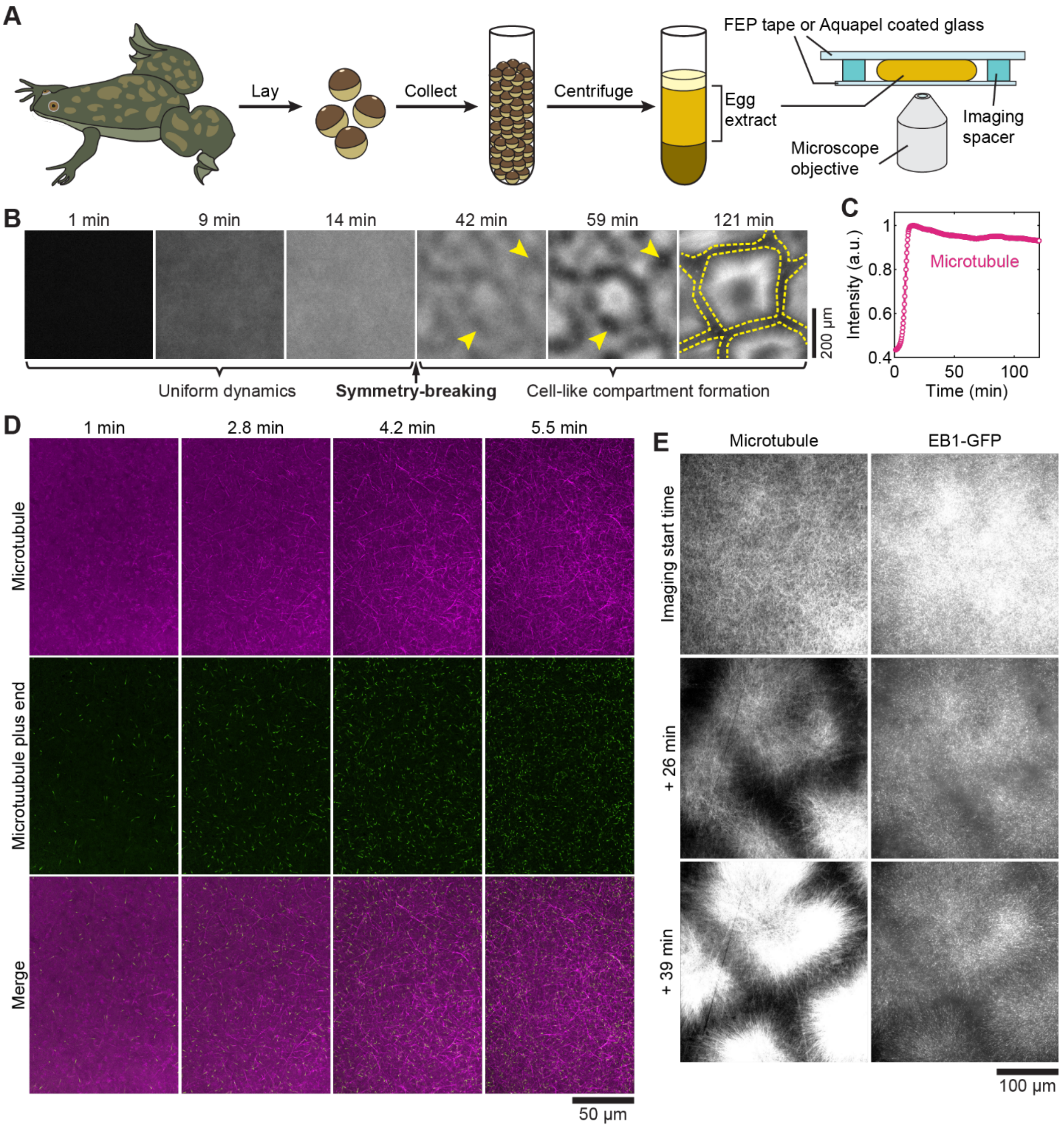

Fig. S1. Microtubule dynamics during the initial stage of self-organization in *Xenopus* egg extracts. (A) Schematic diagram of *Xenopus* egg extract preparation and imaging procedure. (B) Epifluorescence time-lapse montage of microtubule dynamics during cell-like compartment formation. Imaging started at about 1 min after the extracts were moved from an ice-water bath to a room temperature environment. Yellow arrowheads at 42 min indicate exemplary microtubule-depleted regions that had just emerged. Yellow arrowheads at 59 min indicate microtubule-depleted regions where microtubule foci had emerged. Yellow outlines at

Journal of Cell Science • Supplementary information

121 min indicate the periphery of cell-like compartments. (n = 4). (C) Quantification of SiR- tubulin (used to visualize microtubules) epifluorescence intensity for the images from the experiment shown in (B). Each point on the plot indicates the total fluorescence intensity of an image from its corresponding time point. Fluorescence intensity is measured in arbitrary units (a.u.). (D) Single-plane confocal time-lapse montage of microtubule formation. Imaging started at about 1 min after the extracts were moved from an ice-water bath to a room temperature environment. Microtubules were visualized by SiR-tubulin (magenta), and growing microtubule plus ends were visualized by EB1-GFP (Green). (n = 6). (E) Time-lapse single z-plane confocal images of microtubules and EB1-GFP comets. The images show that EB1-GFP was initially ubiquitously present across the view, but as microtubules self-organized into compartments, EB1-GFP also became spatially patterned and mostly localized to the compartments. The dynamics of the process are reported in Movie 4. (n = 7). Related to Fig. 1.

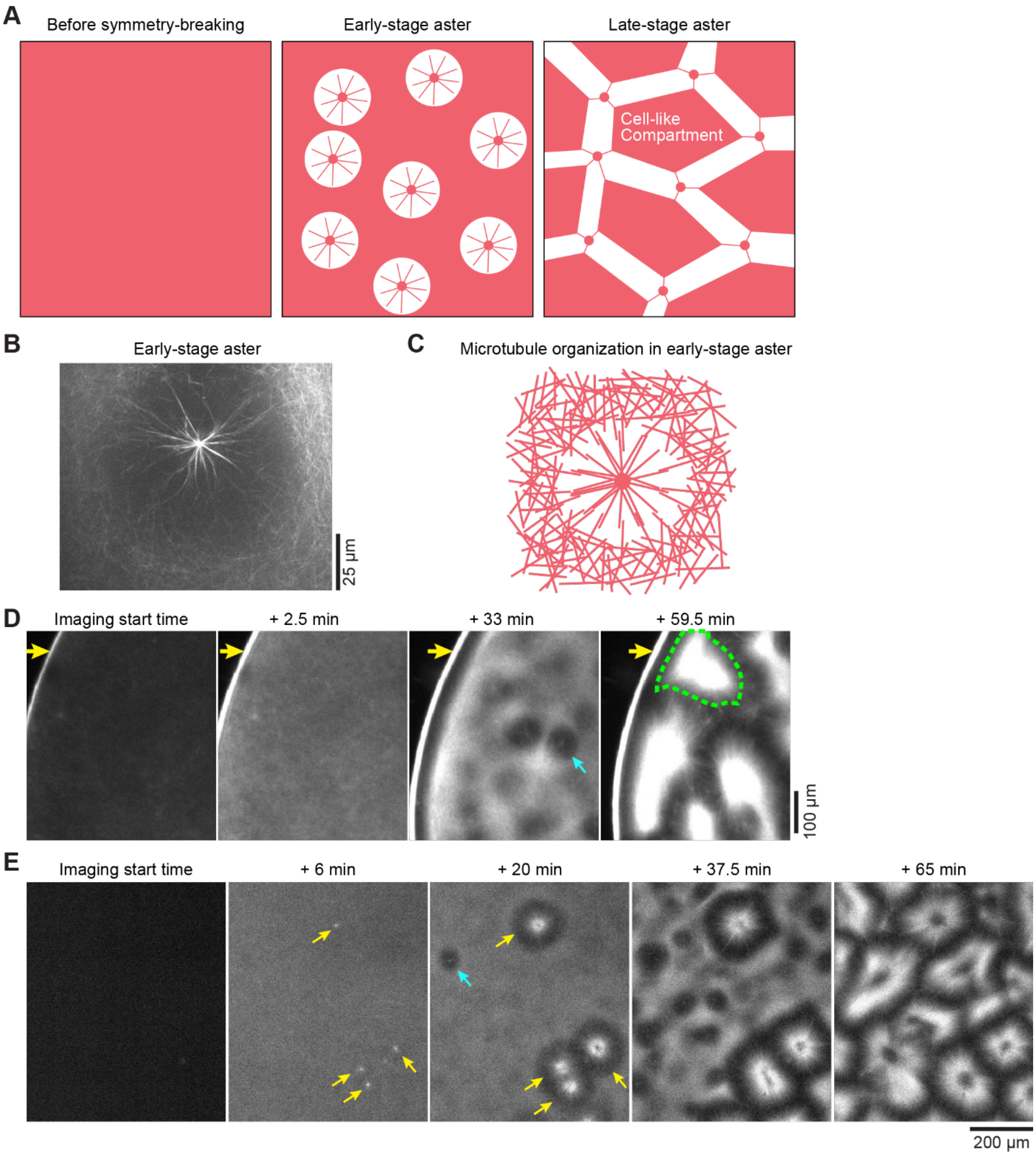

Fig. S2. Features of centrosome-independent asters in *Xenopus* egg extracts. (A) Schematic diagram of aster formation in *Xenopus* egg extracts. (B) A single-plane confocal image of an early-stage aster. The early-stage aster has a radial microtubule array, and it typically emerges in a circular microtubule-depleted zone. (C) A proposed model of microtubule organization in the early-stage aster. (D) Time-lapse widefield epifluorescence images of microtubules in extracts with fresh air supply during incubation. The montage shows that centrosome-independent asters and cell-like compartments formed when oxygen was supplied. The yellow arrows indicate the edge

of the extract droplet, which receded due to evaporation caused by air exchange in the imaging chamber. The cyan arrow indicates an example of centrosome-independent aster. The green dashed line indicates a cell-like compartment. A time-lapse video of the montage is presented in Movie 5. (n = 3). (E) Time-lapse widefield epifluorescence images of microtubules in extracts supplemented with demembranated *Xenopus laevis* sperm nuclei (each was associated with a centrosome). The montage shows that centrosome-independent asters and cell-like compartments formed in extracts containing sperm nuclei. The yellow arrows indicate microtubule asters nucleated by sperm centrosomes. The cyan arrow indicates an example of centrosome-independent aster. Movie 6 presents imagery from the same experiment with a larger field of view. (n = 8). Related to Fig. 1.

Journal of Cell Science • Supplementary information

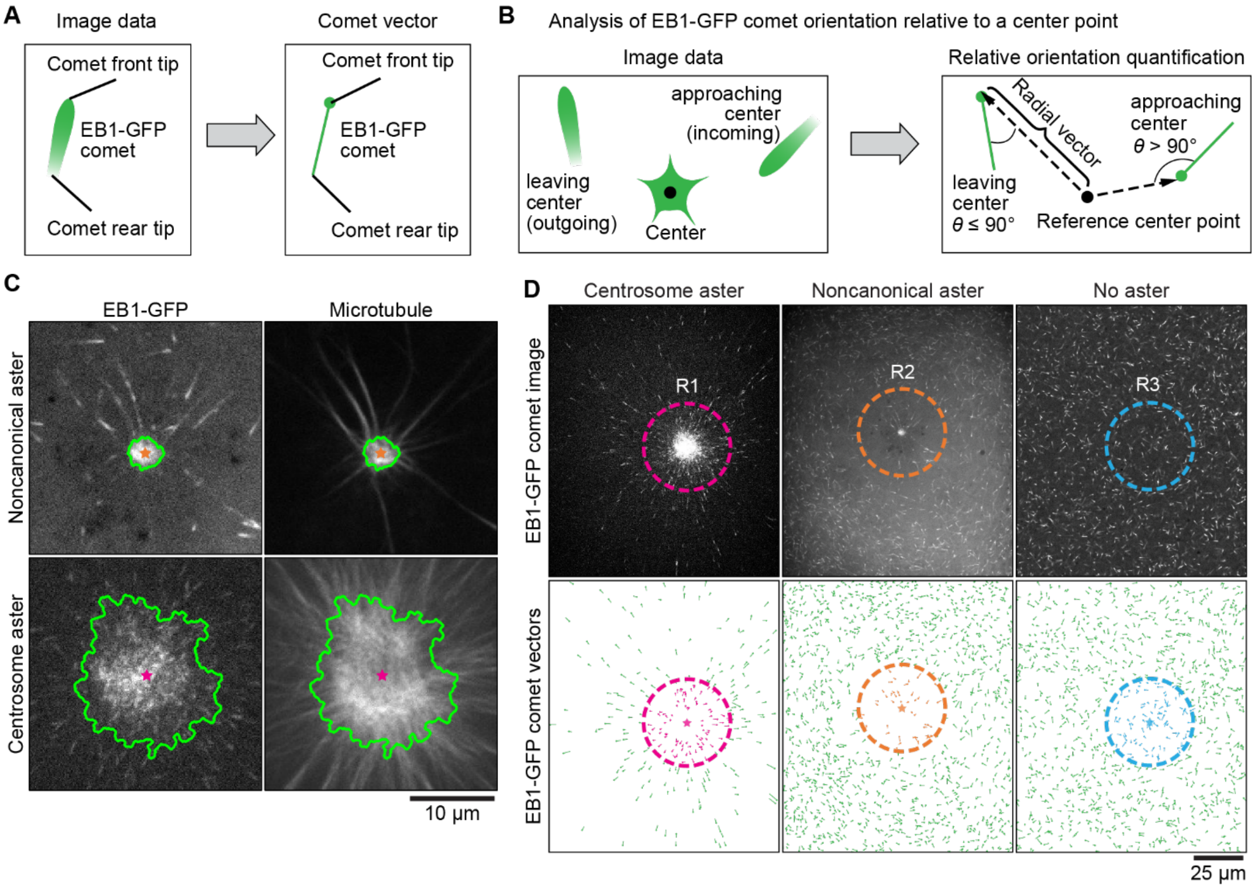

Fig. S3. The method for analyzing microtubule orientation. (A) Schematic diagram for determining the comet vector from an EB1-GFP comet image (See Materials and Methods for details). The comet vector is represented by a green polar line segment, with the vector end point represented by a dot. (B) Schematic diagram for defining the angle metric θ and how θ is used to determine whether the EB1-GFP comet is centripetally or centrifugally orientated relative to a chosen reference center point. The dashed line arrows are radial vectors, and the green segments with a dot as the head are comet vectors (See Materials and Methods for details). (C) Hand-traced image segmentation mask outlines (green lines) of the aster center regions with elevated EB1-GFP signal for the noncanonical aster and centrosome aster (panels in the EB1-GFP column). The traced outlines are superimposed on the confocal images for the microtubule channel of respective asters (panels in the microtubule column). The pentagram stars indicate the geometric centers of the traced regions. (D) Visualization of EB1-GFP comet vectors from confocal images. The comet vector for each EB1-GFP comet is determined following the method described in (A). The top row shows the confocal images of EB1-GFP, and the bottom row shows the corresponding EB1-GFP comet vectors. For the two panels in the centrosome aster column (the first column on the left), the magenta pentagram star in indicates

Journal of Cell Science • Supplementary information

the geometric center of the EB1-GFP enriched region at the aster center, as identified by the procedure described in (C). The magenta dashed circular region centered on the magenta pentagram star encloses the EB1-GFP comets for orientation analysis for the centrosome aster. These comets fall within a 22 μm radius from the center marked by the magenta pentagram star, and their corresponding comet vectors are also shown in magenta in the bottom panel of the column. The other two columns are analogous to the first column, except the color codes are orange and light blue, respectively. For the last column, the pentagram star is chosen at an arbitrary point near the middle of the image. R1, R2, and R3 indicate the regions of comet analysis for centrosome aster, noncanonical aster, and no aster cases, respectively. They are the same color-coded regions marked with R1, R2, and R3 in Fig. 2. Related to Fig. 2.

Journal of Cell Science • Supplementary information

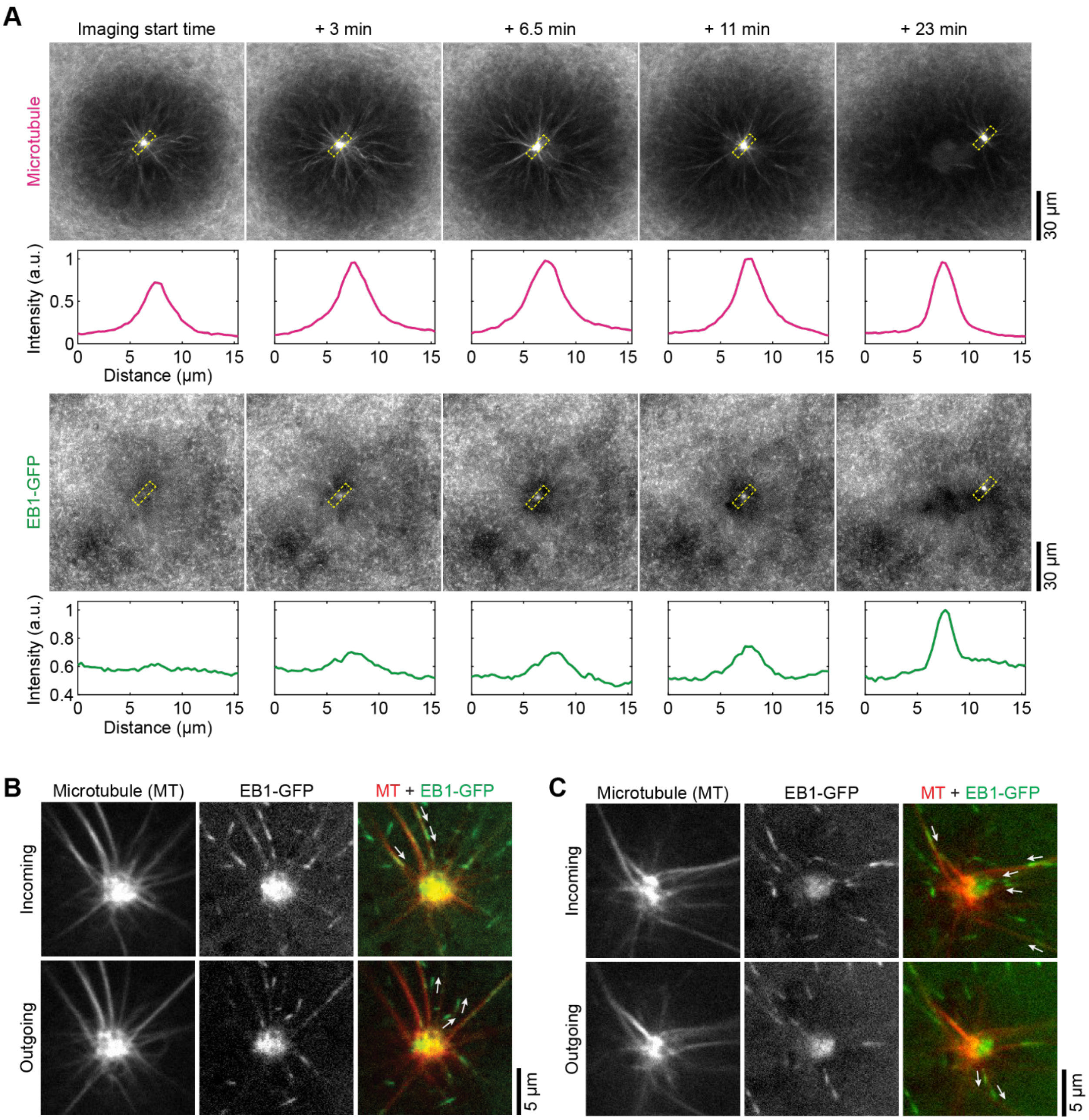

Fig. S4. Features of EB1-GFP near the center of the noncanonical aster. (A) Confocal time- lapse montage of microtubule (top row) and EB1-GFP (third row from the top) dynamics in *Xenopus* egg extracts, showing that EB1-GFP enrichment at the noncanonical aster center occurred after microtubule enrichment. Each image is a maximum intensity projection of 6 confocal planes spanning 15 µm of depth. Images in the same column are from the same view at the same time point (indicated by the text at the top of the column), but from different fluorescence channels. Imaging started at an arbitrary time point shortly after the microtubule aster in view had fully formed. The plot below each image is the fluorescence intensity profile along a 4.8 µm thick, 15.4 µm long line segment (yellow

Journal of Cell Science • Supplementary information

dashed rectangle) that starts at the bottom left and ends at the top right. The line segments (yellow dashed rectangles) from the same column are identically positioned in the microtubule and EB1-GFP images. For each point on the curve in the plot, the horizontal coordinate is the distance from the start of the line segment (the bottom left short edge of the yellow dashed rectangle), and the vertical coordinate is the average fluorescence intensity of the pixels across the width of the line segment at that distance. The intensity units in different fluorescence channels are not comparable. (B) Single-plane confocal images of microtubule and EB1-GFP near the center of an early-stage noncanonical aster, showing examples of EB1-GFP comets coming to and going out of the center. (C) Single-plane confocal images of microtubule and EB1-GFP near the center of a late- stage noncanonical aster, showing examples of EB1-GFP comets coming to and going out of the center. In (B) and (C), the white arrows indicate the direction the comet head is pointing to. The validity of the direction is verified by time-lapse imaging data for each arrow-marked comet. Related to Fig. 2.

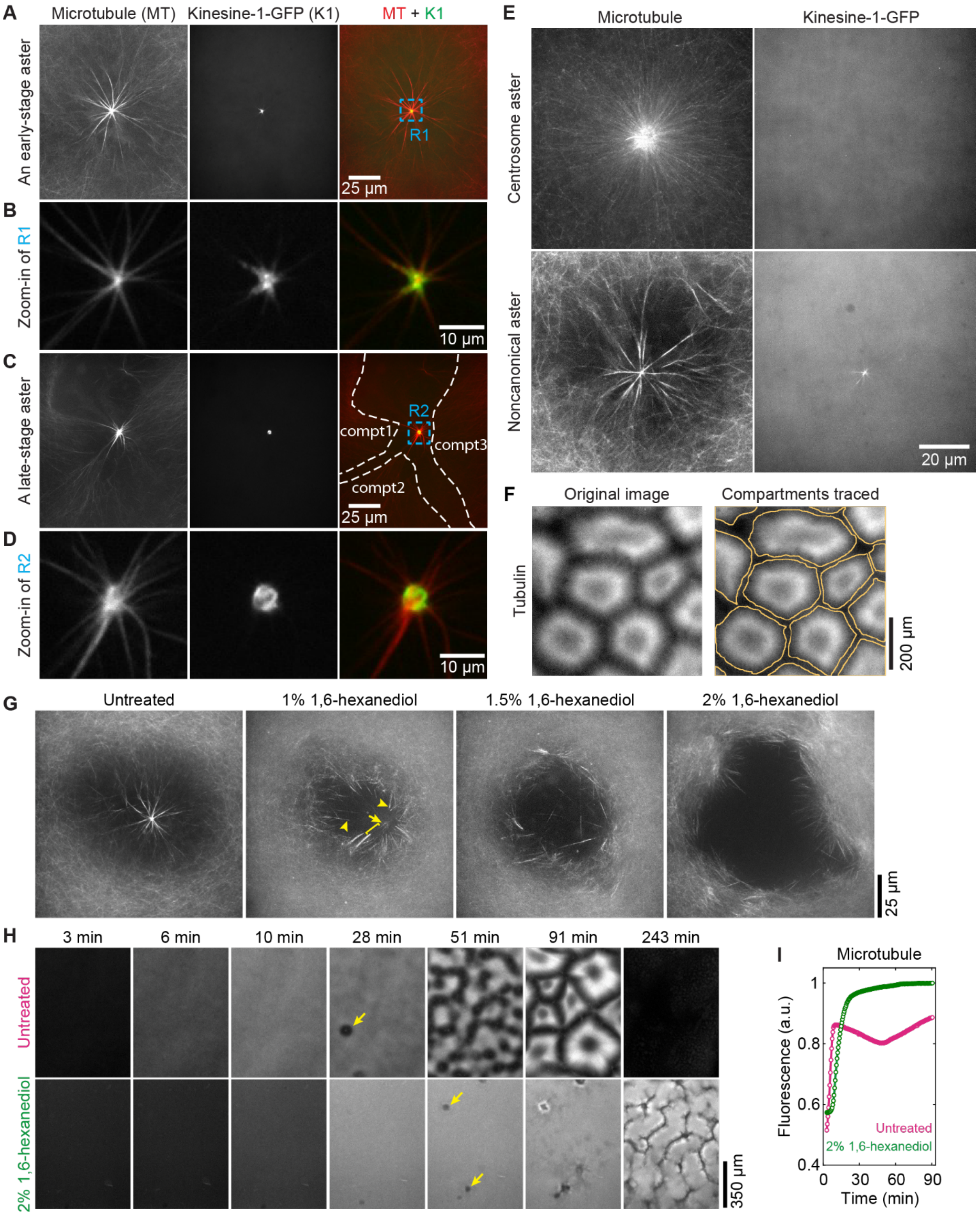

Fig. S5. Kinesin-1-GFP localization in asters, quantification of tubulin fluorescence inside and outside cell-like compartments, and the effect of 1,6-hexanediol on extracts. (A) Single-plane confocal images of an early-stage noncanonical aster, showing accumulation of

Journal of Cell Science • Supplementary information

kinesin-1-GFP at the microtubule organizing center. This is the same aster shown in Fig. 2H. The images provide a wider field of view and show that kinesin-1-GFP is not enriched in regions outside than the aster center. (B) Magnified views of the square region R1 marked in (A), showing the co-localization pattern of kinesin-1-GFP and microtubules. The display ranges of the fluorescence signals for kinesin-1-GFP and microtubules are adjusted to allow subtle features of the co-localization pattern to be more visible compared the Fig. 2H. (C) Single-plane confocal images of a late-stage noncanonical aster, showing accumulation of kinesin-1-GFP at its center. Compt1, compt2 and compt3 indicate 3 different cell-like compartments, each outlined by a white dashed line. (D) Magnified views of the square region R2 marked in (C), showing the co-localization pattern of kinesin-1-GFP and microtubules. (E) Confocal images of microtubule and kinesin-1-GFP localization in a centrosome aster (top row) and a noncanonical aster (bottom row), showing that kinesin-1-GFP accumulated at the center of the noncanonical aster but not that of the centrosome aster. The centrosome aster images are maximum intensity projections spanning 20 μm of z-depth. The noncanonical aster images are maximum intensity projections spanning 4 μm of z-depth. (n = 2). (F) An example microtubule image showing cell-like compartments (left) and the same image with compartment boundaries traced out manually (right, orange lines). Fluorescence signals inside and outside the compartments were calculated based on such manually traced compartment boundaries. (G) Confocal images of extracts treated with various concentrations of 1,6-hexanediol, a chemical that interferes with weak hydrophobic protein-protein interactions. The images are maximum intensity projections of 11, 38, 36, and 14 confocal planes spanning 10 μm, 37 μm, 35 μm, and 13 μm of depth, for the control, 1%, 1.5% and 2% samples, respectively. The depths were chosen to capture representative features in the microtubule-depleted zones. The yellow arrow in the 1% panel indicates the vestige of a severely weakened center. The yellow arrowheads indicate examples of microtubule bundles pointing to the organizing center. The yellow bracket indicates a microtubule bundle connected to the center. (n = 4). (H) Time-lapse montage of microtubule dynamics in untreated and 2% 1,6-hexanediol-treated extracts. Images were acquired by widefield epifluorescence microscopy. Imaging started at about 3 min after the extracts were moved from an ice-water bath to a room temperature environment. The yellow arrows indicate the earliest microtubule-depleted zones in respective samples. At 243 min, the untreated sample was apoptotic, and microtubules had depolymerized, resulting in the loss of SiR-tubulin fluorescence. (n = 3). (I) Quantification of microtubule fluorescence intensity in untreated (magenta) and 2% 1,6-hexanediol-treated (green) extracts from the experiment shown in (H) in arbitrary units (a.u.). Related to Fig. 2 and Fig. 5.

Journal of Cell Science • Supplementary information

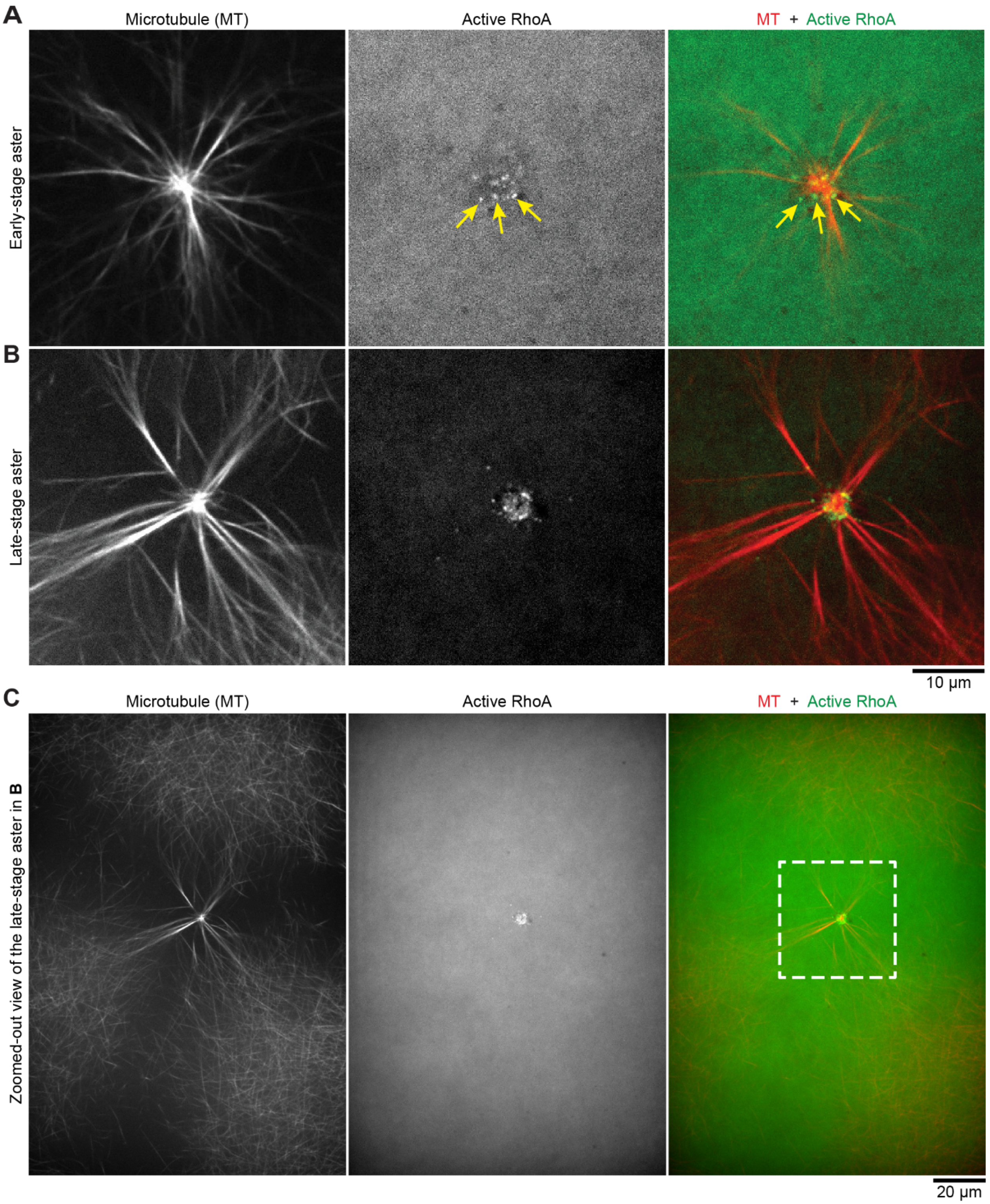

Fig. S6. The noncanonical aster accumulates active RhoA at its center. (A) Single-plane confocal images of an early-stage noncanonical aster, showing accumulation of active RhoA at the microtubule organizing center. The yellow arrows indicate active RhoA puncta. (B) Single-plane confocal images

Journal of Cell Science • Supplementary information

of a late-stage noncanonical aster, showing accumulation of active RhoA at the microtubule organizing center. (C) Single-plane confocal images offering a large-field view of the late-stage aster shown in (B), showing that RhoA activity was restricted to the center of the noncanonical aster. The late-stage aster is located at a corner where 3 cell-like compartments meet. The white dashed square region marks the exact location of the late-stage aster panels shown in (B). The panels in (A) and (B) share the scale bar in (B). Related to Fig. 6.

Journal of Cell Science • Supplementary information

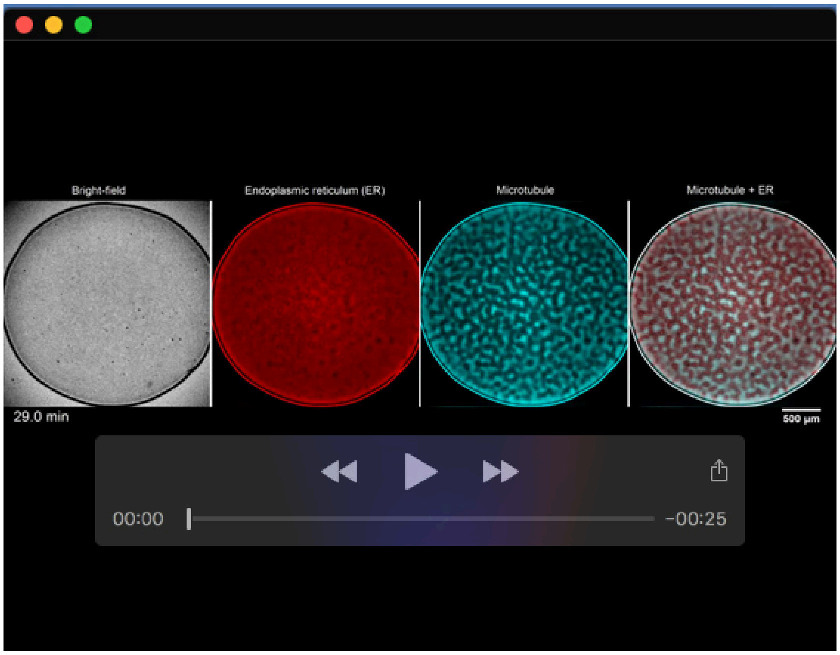

Movie 1. Self-organization of cell-like compartments in *Xenopus* egg extracts. Imaged with a widefield epifluorescence microscope and a 5x objective. Time stamp 0 corresponds to the imaging start time. Related to Fig. 1.

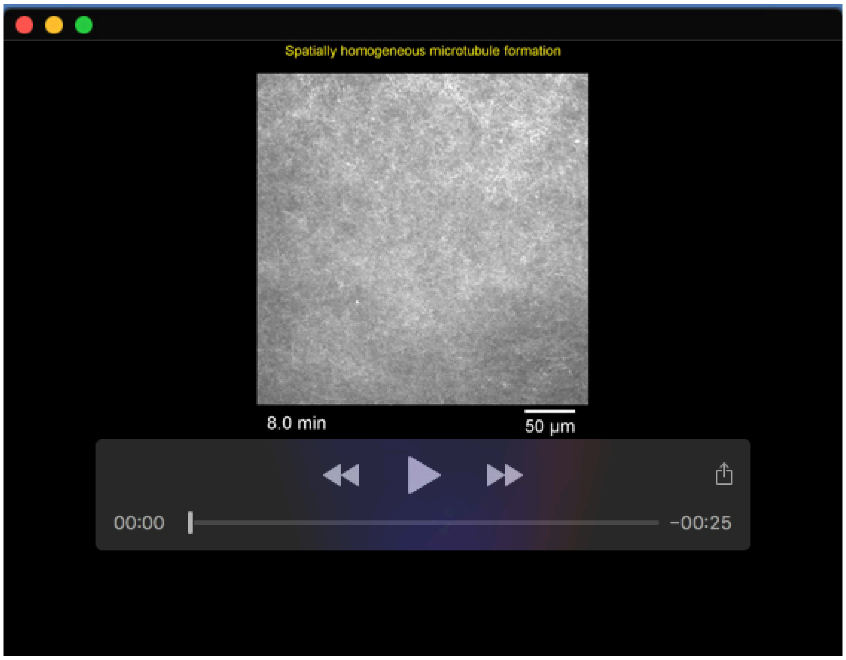

Movie 2. Confocal time-lapse video of cell-like compartment formation in *Xenopus* egg extracts. Imaged with a confocal microscope and a 20x air objective. This movie comprises the complete set of time series images from the experiment shown in Fig. 1B. It shows key events and structures in the compartment formation process. Each frame is a maximum intensity projection of 17 confocal planes spanning 32 µm of depth. Imaging started 3 min after the extract had commenced room temperature incubation, and therefore the time stamps started from 3 min. Related to Fig. 1.

Journal of Cell Science • Supplementary information

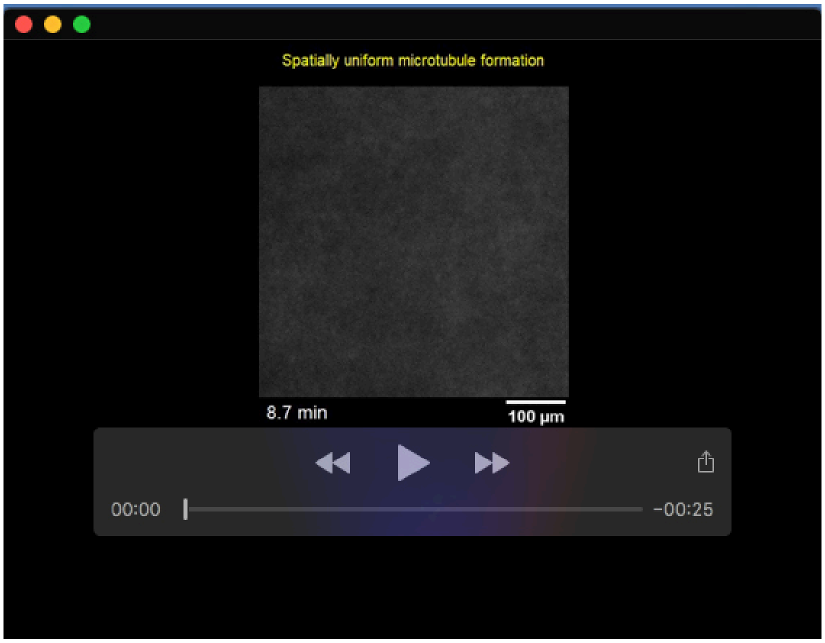

Movie 3. Widefield time-lapse video of cell-like compartment formation in *Xenopus* egg extracts. Imaged with a widefield epifluorescence microscope and a 5x objective. This movie comprises the complete set of time series images from the experiment shown in Fig. S1B. It shows key events and structures in the compartment formation process. Imaging started 1 min after the extract had commenced room temperature incubation, and therefore the time stamps started from 1 min. Related to Fig. 1.

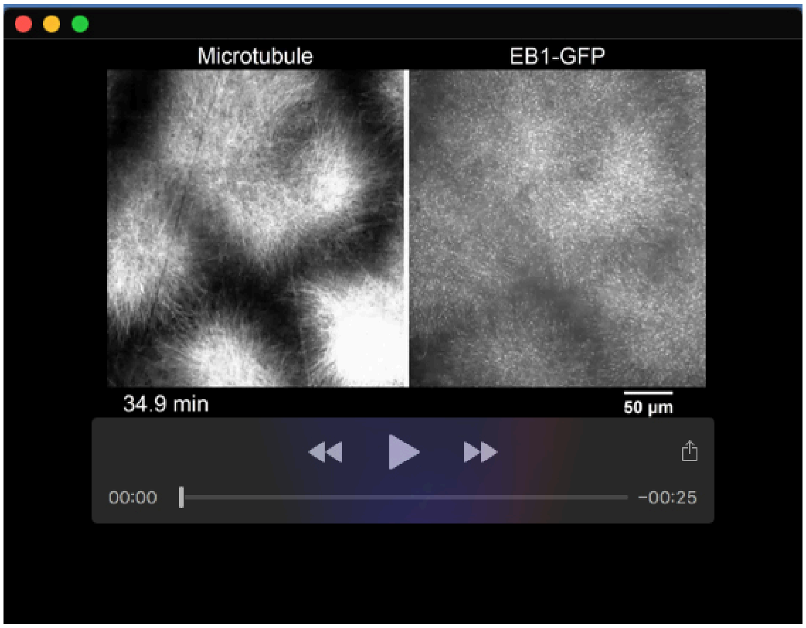

Movie 4. Symmetry-breaking in microtubule and EB1-GFP spatial patterns during cell-like compartment formation. The movie shows that microtubules and EB1-GFP comets were initially spatially uniformly present, but as microtubules subsequently self-organized into cell-like compartments EB1-GFP also became spatially patterned, with most of the comets appearing inside the compartments. The images capture a single confocal plane very close to the coverslip to ensure clear EB1-GFP signals. Related to Fig. 1.

Journal of Cell Science • Supplementary information

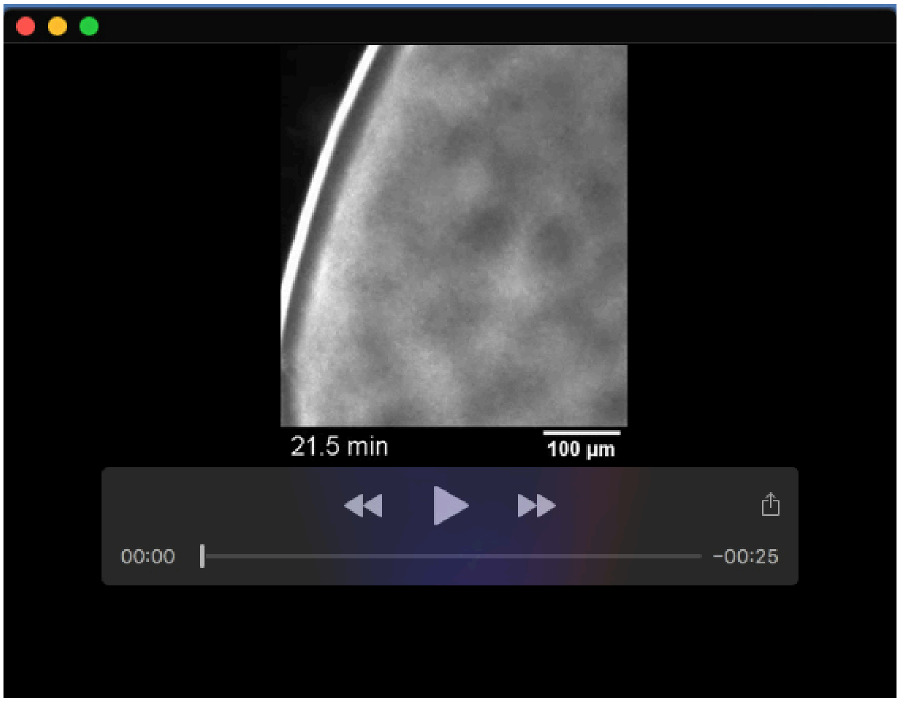

Movie 5. Noncanonical asters and cell-like compartments form normally when oxygen is supplied to the extracts. Widefield time-lapse images of microtubule dynamics in egg extracts supplied with fresh air during incubation, showing noncanonical aster and cell-like compartment formation in the extracts. Related to Fig. 1.

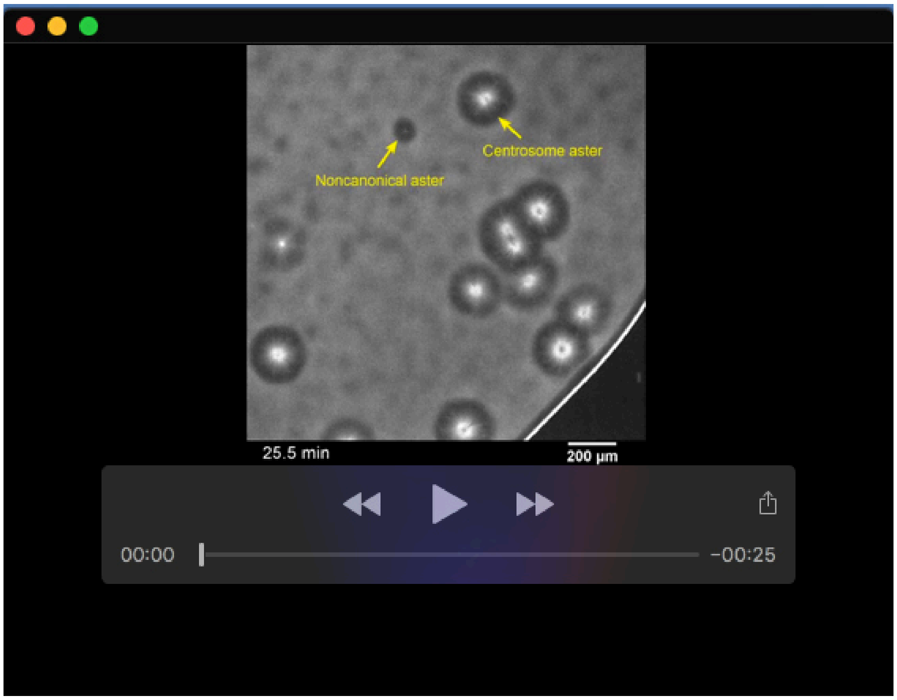

Movie 6. Noncanonical asters and cell-like compartments form normally in extracts containing *Xenopus laevis* sperm nuclei. Widefield time-lapse images of microtubule dynamics in egg extracts supplemented with demembranted sperm nuclei, showing noncanonical aster and cell-like compartment formation in the extracts. Related to Fig. 1.

Journal of Cell Science • Supplementary information

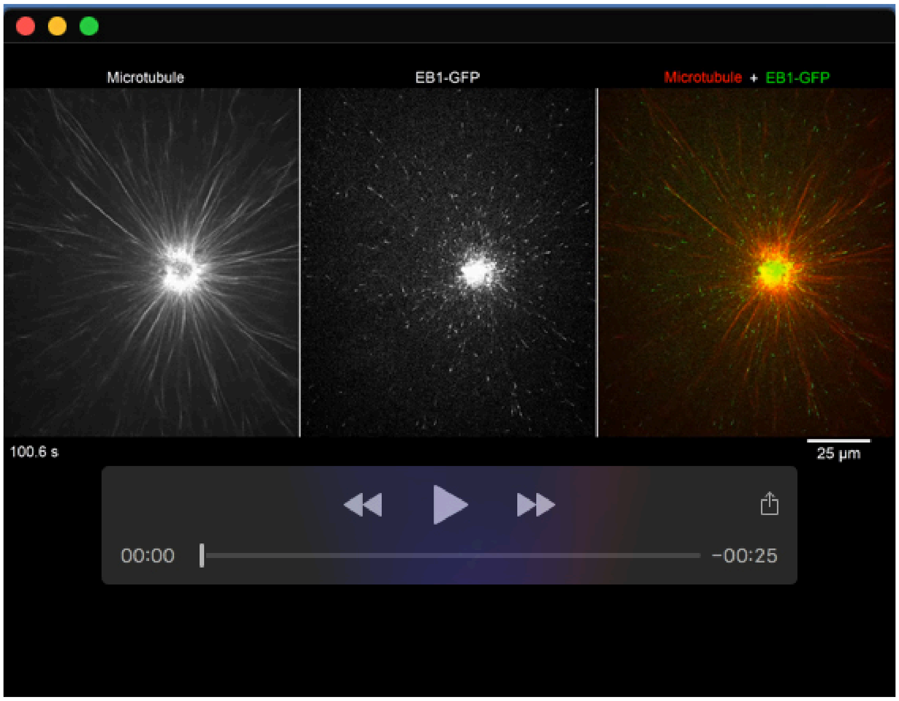

Movie 7. Microtubule and EB1-GFP dynamics in a centrosome aster assembled in *Xenopus* egg extracts. The movie shows that EB1-GFP comets near the aster center mostly move away from the aster center. The movie is a time-lapse of single-plane confocal images from the same experiment shown in Fig. 2. Time stamp 0 corresponds to the imaging start time. Related to Fig. 2.

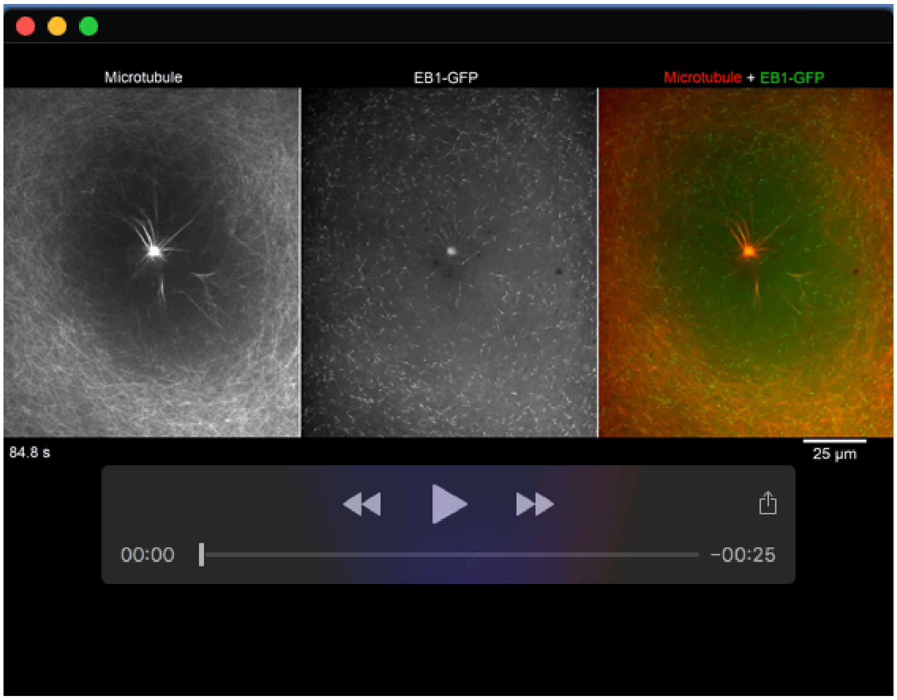

Movie 8. Microtubule and EB1-GFP dynamics in a noncanonical aster assembled in *Xenopus* egg extracts. The movie shows that EB1-GFP comets near the aster center mostly move towards the aster center. The movie is a time-lapse of single-plane confocal images from the same experiment shown in Fig. 2. Time stamp 0 corresponds to the imaging start time. Related to Fig. 2.

Journal of Cell Science • Supplementary information

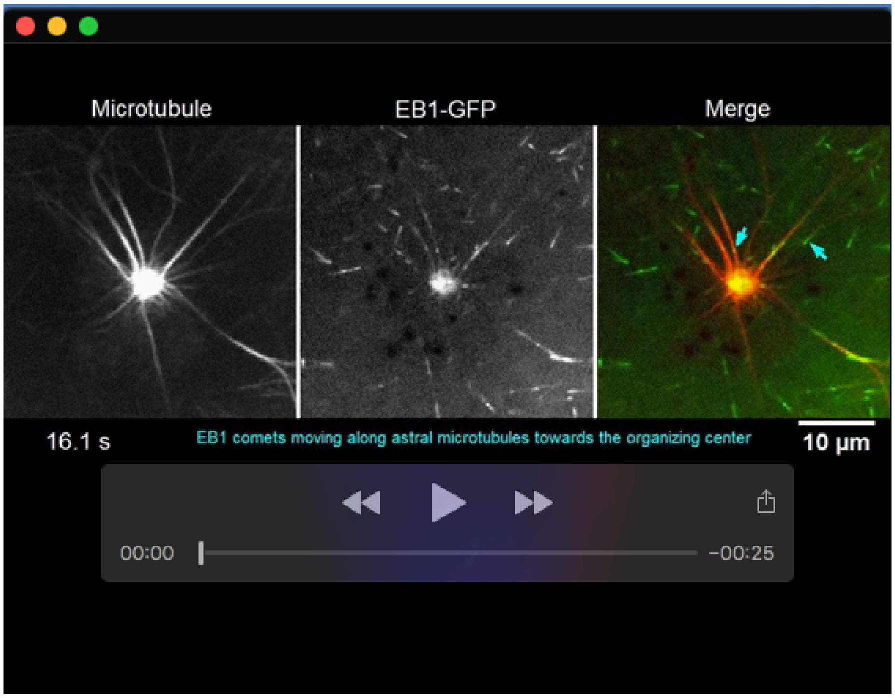

Movie 9. Detailed microtubule and EB1-GFP dynamics in a noncanonical aster assembled in *Xenopus* egg extracts. This movie shows examples of EB1-GFP comets that go into and come out of the microtubule organizing center, where EB1-GFP is enriched. The movie is a time-lapse of single-plane confocal images from the same experiment shown in Fig. 2. Time stamp 0 corresponds to the imaging start time. Related to Fig. 2.

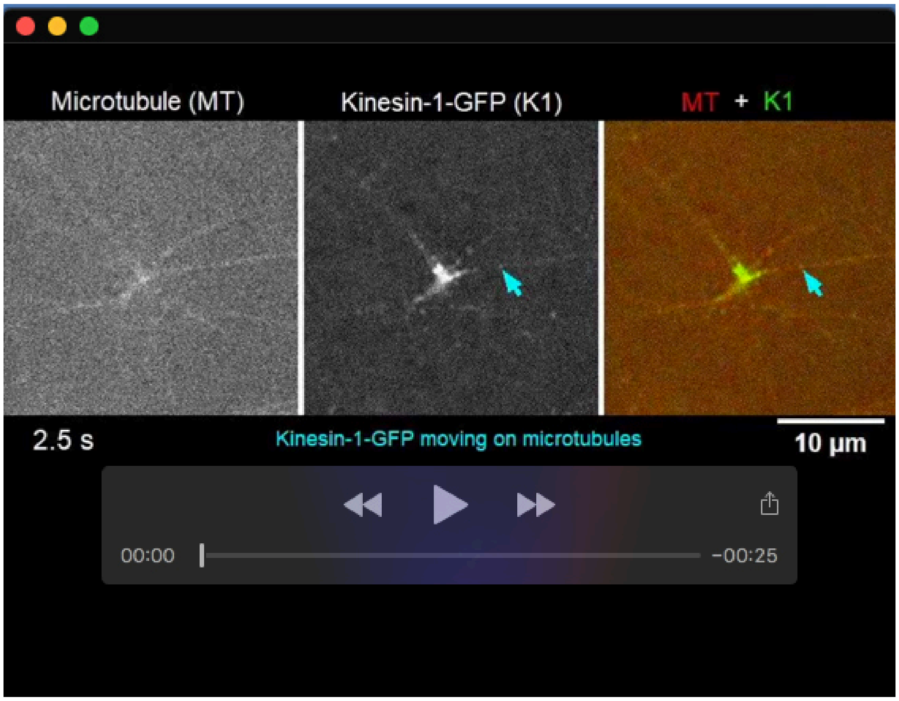

Movie 10. Microtubule and kinesin-1-GFP dynamics in *Xenopus* egg extracts. This movie shows that human kinesin-1-GFP moves along microtubules, indicating that they are functioning normally. The movie is a time-lapse of single-plane confocal images. Time stamp 0 corresponds to the imaging start time. Related to Fig. 2.

Journal of Cell Science • Supplementary information

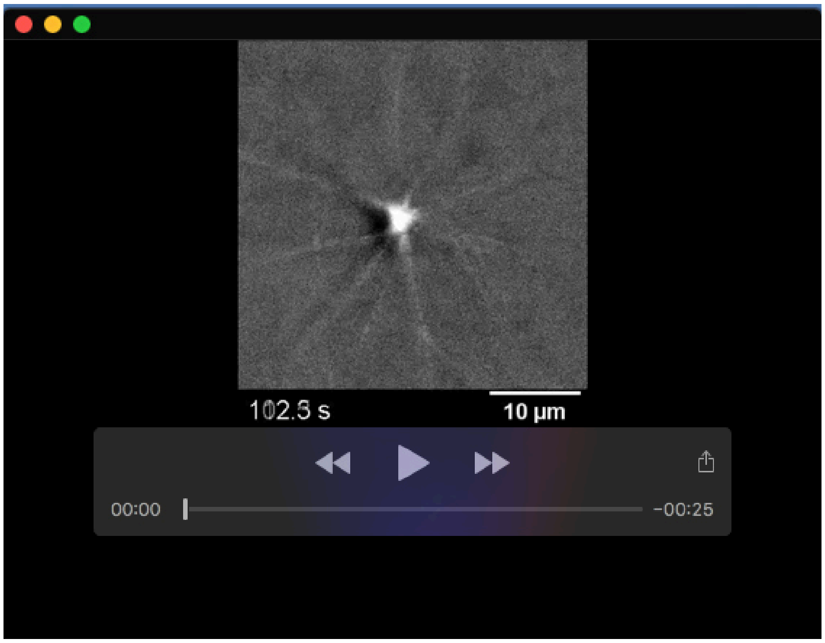

Movie 11. Kinesin-1-GFP dynamics in the noncanonical aster. This movie shows the dynamics of human kinesin-1-GFP in a noncanonical aster. The movie is a time-lapse of confocal images from the same experiment as Fig. 2H. Each frame in the movie is a sum-of- slices projection of 3 confocal planes spanning 2 µm of depth. Time stamp 0 corresponds to the imaging start time. Related to Fig. 2.

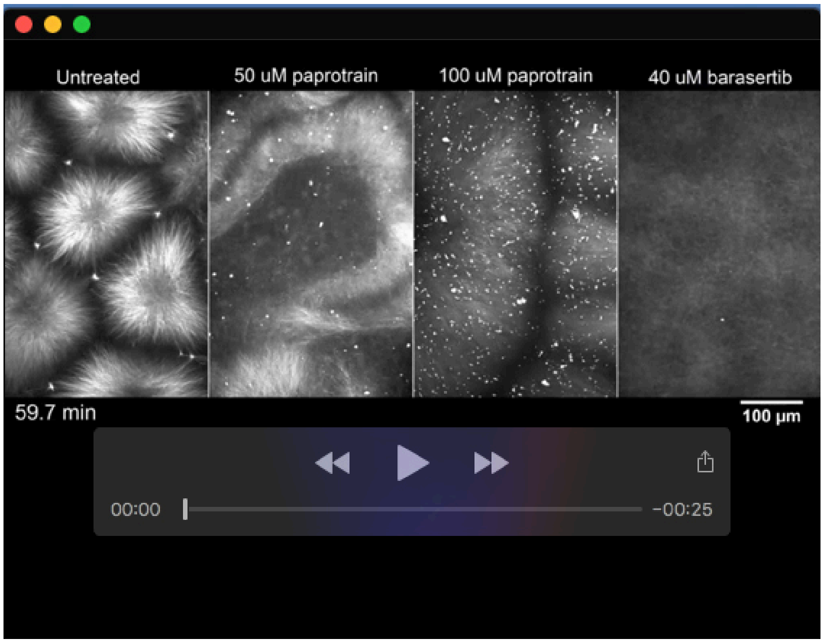

Movie 12. Noncanonical aster formation requires MKLP2 and Aurora kinase B. Time-lapse video of microtubule dynamics in control extracts and extracts treated with 50 µM paprotrain (MKLP2 inhibitor), 100 µM paprotrain, and 40 µM barasertib (Aurora kinase B inhibitor). Noncanonical asters formed normally in control extracts but not MKLP2 or Aurora kinase B inhibited extracts. Cell-like compartments formed in control and Aurora kinase B inhibited extracts but not in MKLP2 inhibited extracts. Related to Fig. 3.

Journal of Cell Science • Supplementary information

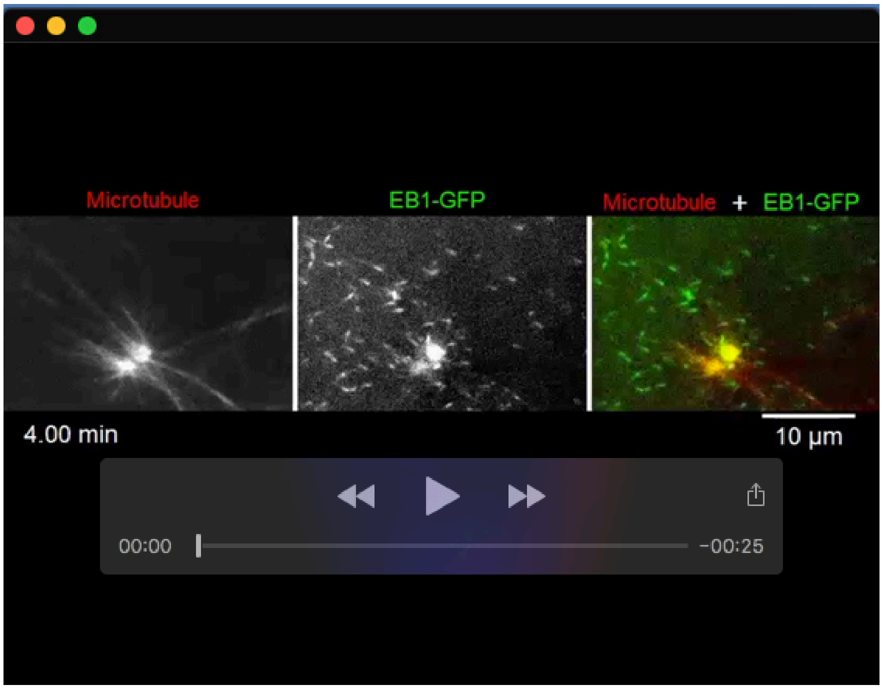

Movie 13. Microtubule and EB1-GFP dynamics in spontaneously merging noncanonical asters in egg extracts. The movie is a time-lapse of confocal images from the same experiment shown in Fig. 4A, each frame is a maximum intensity projection of 4 confocal planes spanning 6 μm of depth. Time stamp 0 corresponds to the imaging start time. Related to Fig. 4.

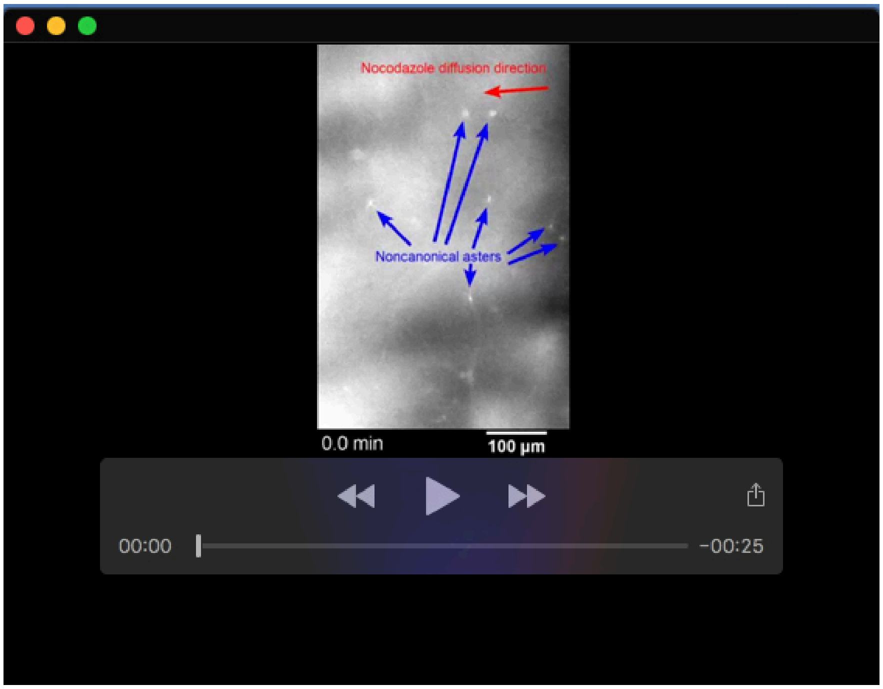

Movie 14. The noncanonical aster is more resistant to microtubule depolymerization than regular microtubules. Widefield epifluorescence time-lapse video of microtubule dynamics in an extract droplet treated with localized nocodazole administration to the right of the field of view. Diffusion of nocodazole, a microtubule polymerization inhibitor, depolymerized regular microtubules across the field of view, but the noncanonical asters persisted much longer. Related to Fig. 5.

Journal of Cell Science • Supplementary information

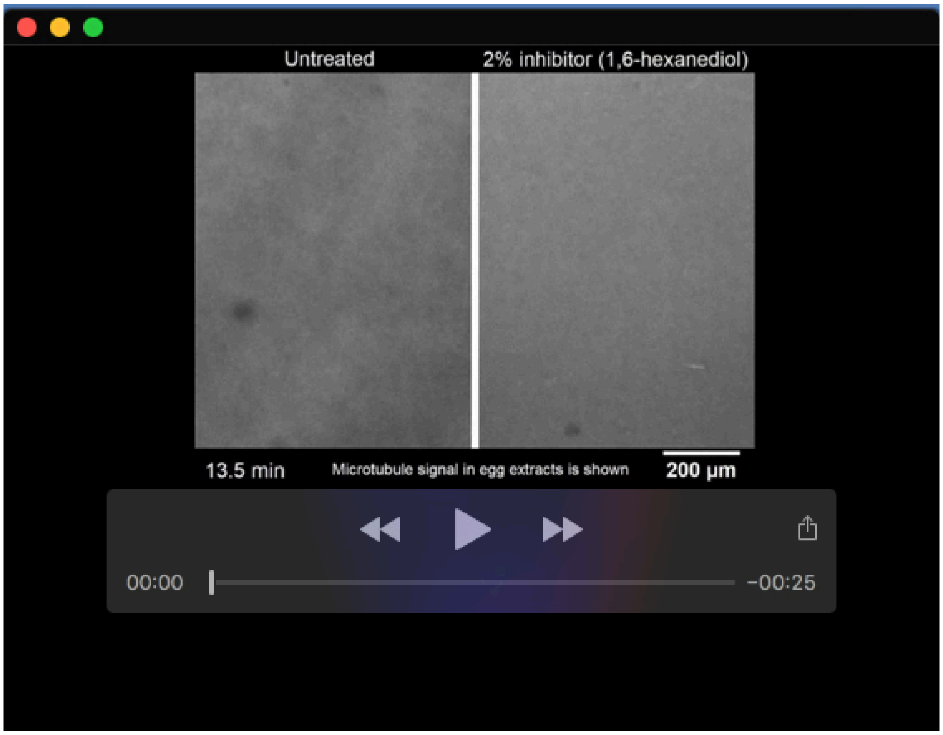

Movie 15. Microtubule dynamics and patterning in untreated control extracts and extracts treated with 2% (w/v) of 1,6-hexanediol. Related to Fig. S5.

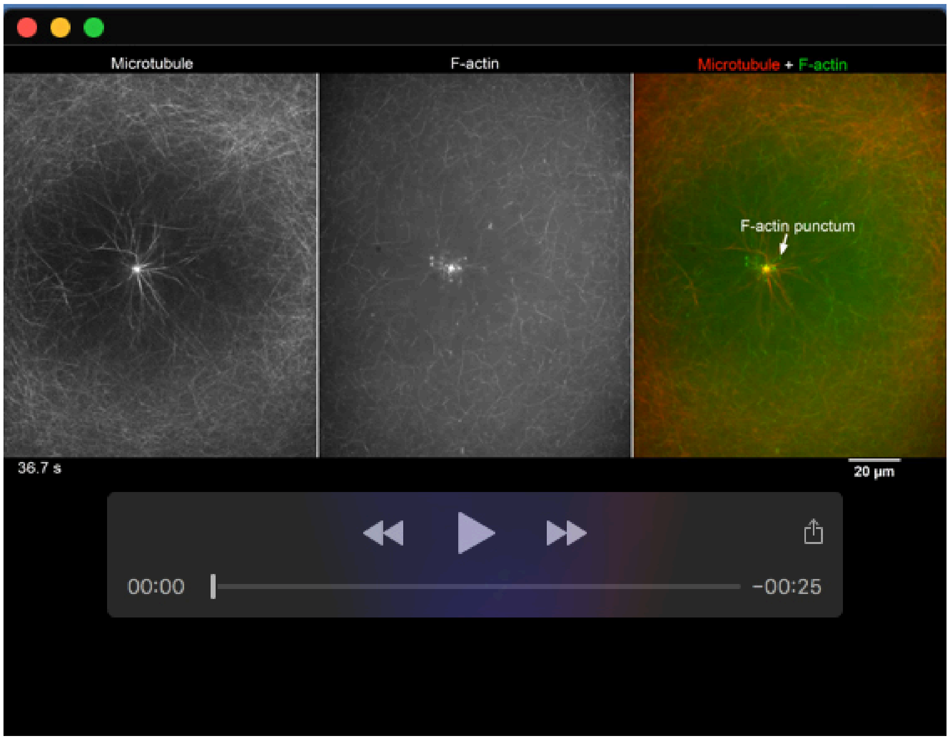

Movie 16. F-actin trafficking in the noncanonical aster. The movie shows that pre-existing F- actin moves toward and accumulates at the center of the noncanonical aster. The movie is a time-lapse of single-plane confocal images. Related to Fig. 6.

Journal of Cell Science • Supplementary information
